# Risk stratified monitoring for methotrexate toxicity in immune mediated inflammatory diseases: prognostic model development and validation using primary care data from the UK

**DOI:** 10.1136/bmj-2022-074678

**Published:** 2023-05-30

**Authors:** Georgina Nakafero, Matthew J Grainge, Hywel C Williams, Tim Card, Maarten W Taal, Guruprasad P Aithal, Christopher P Fox, Christian D Mallen, Danielle A van der Windt, Matthew D Stevenson, Richard D Riley, Abhishek Abhishek

**Affiliations:** 1 Academic Rheumatology, University of Nottingham, Nottingham NG5 1PB, UK; 2 Lifespan and Population Health, School of Medicine, University of Nottingham, Nottingham, UK; 3 Centre for Kidney Research and Innovation, Translational Medical Sciences, University of Nottingham, Derby, UK; 4 Nottingham Digestive Diseases Centre, Translational Medical Sciences, University of Nottingham, Nottingham, UK; 5 Department of Haematology, Nottingham University Hospital NHS Trust, Nottingham, UK; 6 Primary Care Centre Versus Arthritis, School of Medicine, Keele University, Keele, UK; 7 School of Health and Related Research, University of Sheffield, Sheffield, UK; 8 Institute of Applied Health Research, College of Medical and Dental Sciences, University of Birmingham, Birmingham, UK

## Abstract

**Objective:**

To develop and validate a prognostic model to inform risk stratified decisions on frequency of monitoring blood tests during long term methotrexate treatment.

**Design:**

Retrospective cohort study.

**Setting:**

Electronic health records within the UK’s Clinical Practice Research Datalink (CPRD) Gold and CPRD Aurum.

**Participants:**

Adults (≥18 years) with a diagnosis of an immune mediated inflammatory disease who were prescribed methotrexate by their general practitioner for six months or more during 2007-19.

**Main outcome measure:**

Discontinuation of methotrexate owing to abnormal monitoring blood test result. Patients were followed-up from six months after their first prescription for methotrexate in primary care to the earliest of outcome, drug discontinuation for any other reason, leaving the practice, last data collection from the practice, death, five years, or 31 December 2019. Cox regression was performed to develop the risk equation, with bootstrapping used to shrink predictor effects for optimism. Multiple imputation handled missing predictor data. Model performance was assessed in terms of calibration and discrimination.

**Results:**

Data from 13 110 (854 events) and 23 999 (1486 events) participants were included in the development and validation cohorts, respectively. 11 candidate predictors (17 parameters) were included. In the development dataset, the optimism adjusted R^2^ was 0.13 and the optimism adjusted Royston D statistic was 0.79. The calibration slope and Royston D statistic in the validation dataset for the entire follow-up period was 0.94 (95% confidence interval 0.85 to 1.02) and 0.75 (95% confidence interval 0.67 to 0.83), respectively. The prognostic model performed well in predicting outcomes in clinically relevant subgroups defined by age group, type of immune mediated inflammatory disease, and methotrexate dose.

**Conclusion:**

A prognostic model was developed and validated that uses information collected during routine clinical care and may be used to risk stratify the frequency of monitoring blood test during long term methotrexate treatment.

## Introduction

Low dose methotrexate, administered weekly, is the first line glucocorticoid sparing drug used to treat immune mediated inflammatory diseases such as rheumatoid arthritis, psoriatic arthritis, and cutaneous psoriasis resistant to topical treatment or phototherapy.^1^ ^2^ ^3^ ^4^ ^5^ Methotrexate is also used in the treatment of steroid dependent inflammatory bowel disease and is often combined with biological medicines to optimise efficacy and prevent the formation of antidrug antibodies.^6^ ^7^ ^8^ Methotrexate use has increased in the era of biologics, with only a few patients (about 3%) with rheumatoid arthritis or with psoriasis with or without arthritis prescribed biologics in European countries.^9^ ^10^ ^11^ ^12^

Although effective and well tolerated, methotrexate can cause cytopenia, raised liver enzyme levels, and acute kidney injury, mostly during the first few months of treatment.^13^ ^14^ Concern about these side effects has led to the recommendation to perform blood tests every two to four weeks to monitor patients for full blood count, liver function, and urea electrolytes and creatinine during the initial few months of treatment, followed thereafter by tests every three months for everyone.^15^ ^16^ However, no evidence base supports the use of three monthly blood tests in the early detection of liver, blood, or kidney toxicity during long term methotrexate treatment.

We previously reported that clinically significant cytopenia, raised liver enzyme levels, and acute kidney injury that required methotrexate discontinuation were infrequent after the first year of methotrexate treatment.^17^ Indeed, many abnormalities detected during the monitoring of blood tests for long term methotrexate treatment are subsequently found to be due to an intercurrent illness or its treatment. Nevertheless, the detection of these abnormalities causes anxiety, requires repeat testing, and may result in treatment interruption that results in a disease flare-up. Unnecessary blood tests waste patient’s time and healthcare resources, including the time of general practitioners and phlebotomists.

It would be beneficial to predict the risk of clinically significant abnormal blood test results during long term methotrexate treatment to inform the frequency of testing for individuals. To better understand this risk, we developed and validated a prognostic model for estimating the probability of clinically significant methotrexate toxicity during long term treatment and stratifying those at greatest and least risk.

## Methods

In this retrospective cohort study performed from 1 January 2007 to 31 December 2019, we used data from the Clinical Practice Research Datalink (CPRD) Gold and CPRD Aurum for model development and validation, respectively.^18^ ^19^ CPRD is an anonymised longitudinal database of electronic health records originating during clinical care in the NHS. As CPRD has almost universal coverage of UK residents, participants who contribute information to the database are representative of the UK population.^18^ CPRD includes information on demographic factors, lifestyle factors (eg, smoking status, alcohol intake), diagnoses, blood test results, and prescriptions issued in primary care. As CPRD Gold and CPRD Aurum use different software packages for data capture, they complement each other for coverage of general practices. A bridging file provided by CPRD was used to identify those general practices that contributed data to both databases, and these practices were only included in the development cohort.

This study was reported in line with the transparent reporting of a multivariable prediction model for individual prediction or diagnosis (TRIPOD) guidelines.^20^

### Study population

Adults aged 18 years or older with a new diagnosis of an immune mediated inflammatory disease (eg, rheumatoid arthritis, axial spondyloarthritis, psoriasis with or without arthritis, and inflammatory bowel disease) and prescribed methotrexate by their GP were eligible for study inclusion. Patients were required to have no immune mediated inflammatory disease recorded for at least one year with their current general practice to be classified as having a new diagnosis of immune mediated inflammatory disease.^21^ ^22^ This minimised the chance of patients who had used long term methotrexate for established immune mediated inflammatory disease appearing as new users of methotrexate when they moved to a different general practice.^21^ ^22^ Additionally, it was required that patients receive their first methotrexate prescription either after the first record of immune mediated inflammatory disease in CPRD or in the preceding 90 days. This 90 day period was chosen because the recording of diagnoses may lag behind prescriptions.

Before the start of follow-up, we excluded patients with severe chronic liver disease, chronic kidney disease stage 4 or 5, or severe haematological diseases. Because methotrexate is contraindicated for these conditions and there would have been substantial uncertainty in outcome ascertainment (see supplementary material for Read code list).

### Methotrexate prescriptions

In the United Kingdom, the initial prescription for methotrexate and dose escalation occurs in hospital out-patient clinics. During this period, hospital specialists organise the monitoring of blood tests and respond to abnormal results. Once a patient’s treatment is established—that is, a stable, well tolerated, and effective methotrexate dose is reached, typically about six months after the start of treatment, GPs take on the responsibility of prescribing for and monitoring patients with periodic blood tests according to NHS shared care protocols.^15^ ^23^ ^24^ During shared care prescribing, GPs seek advice about side effects from hospital specialists, including abnormal blood test results, and the hospital specialists direct changes to treatment.

### Start of follow-up

Patients were followed-up from 180 days after their first prescription for methotrexate in primary care to the earliest of outcome, drug discontinuation for any reason, leaving the practice, last data collection from the practice, death, five years, or 31 December 2019.

### Outcome

The outcome of interest was methotrexate toxicity associated drug discontinuation, defined as a prescription gap of ≥90 days with either an abnormal blood test result or a diagnostic code for an abnormal blood test result within ±60 days of the last prescription date.^17^ The thresholds for abnormal blood test results were: total leucocyte count <3.5×10^9^/L, neutrophil count <1.6×10^9^/L, platelet count <140×10^9^/L, alanine transaminase and/or aspartate transaminase >100 IU/mL, and decline in kidney function, defined as either progression of chronic kidney disease based on medical codes recorded by the GP, or >26 μmol/L increase in creatinine concentration, the threshold for consideration of acute kidney injury.^15^ ^25^ In our previous validation study, only 5.4% of abnormal blood test results in this time window were potentially explained by another illness.^17^

### Predictor selection

Clinical members of the study team suggested predictors based on their clinical expertise and knowledge of the literature.

We included age, sex, body mass index (BMI), alcohol intake, and diabetes because they are associated with drug induced liver injury, and current smoking because it is associated with non-response to methotrexate, potentially requiring higher doses.^26^ ^27^ We included chronic kidney disease because it reduces methotrexate clearance,^28^ and type of immune mediated inflammatory disease because patients with psoriasis are at higher risk of raised liver enzyme levels with methotrexate treatment than patients with rheumatoid arthritis.^17^

Methotrexate dose was included to account for potential dose-dependent toxicity. We also included statins, non-steroidal anti-inflammatory drugs, aspirin (≥300 mg/day), paracetamol (acetaminophen), proton pump inhibitors, carbamazepine, levetiracetam, and valproate as their use is associated with methotrexate toxicity according to the British National Formulary. Hydroxychloroquine was included as it increases the bioavailability of methotrexate.^29^ Sulfasalazine, 5-acetylsalicylate, and other immunosuppressant drugs were included as they can cause cytopenia, raised liver enzyme levels, and acute kidney injury.

Either cytopenia (neutrophil count <2×10^9^/L, total leucocyte count <4×10^9^/L, or platelet count <150×10^9^/L) or raised transaminase (alanine transaminase and/or aspartate transaminase >35 IU/L) levels during the first six months of the methotrexate prescription (ie, before the start of follow-up), were included as prognostic factors because in other studies they predicted cytopenia and/or transaminitis.^30^ ^31^

We used the latest record of demographic and lifestyle factors, diseases recorded within two years before start of follow-up, and latest primary care prescription within six months before start of follow-up to define predictors, except for chronic kidney disease stage 3, which was defined using both GP records and estimated glomerular filtration rate (eGFR) 30-59 mL/min. GPs typically review patients with long term conditions annually. We utilised a look-back period of two years to minimise the risk of missing data from those patients who did not attend in the previous year.

### Sample size

For model development, we used Riley and colleagues’ formulae.^32^ Using those formulae we determined that to minimise model overfitting (a target shrinkage factor of 0.9) and ensure precise estimation of overall risk, we required a minimum sample size of 1398 participants (189 outcomes) based on a maximum of 20 parameters, Cox-Snell R^2^ value of 0.12, estimated outcome rate of 0.057 per person year, and a five year time horizon, and a mean follow-up period of 2.36 years using the findings from our earlier work.^17^ The sample size for external model validation was larger than the typically recommended minimum sample size of 200 events.

### Statistical analysis

Multiple imputation handled missing predictor data on BMI, alcohol intake, and methotrexate dose using chained equations.^33^ We carried out 10 imputations in the development dataset and five imputations in the validation dataset—a pragmatic approach considering the larger size of CPRD Aurum. The imputation model included all candidate predictors, Nelson-Aalen cumulative hazard function, and outcome variables.

#### Model development

Fractional polynomial regression (first order) analysis was used to model non-linear risk relationships with continuous predictors and was found to be no better than the linear terms (P>0.05, comparing models of linear terms with the best fitting first order polynomials), hence continuous predictors were not transformed. All 11 candidate predictors (17 parameters) were included in the Cox model, and the coefficients of each predictor were estimated and combined using Rubin’s rule across the imputed datasets. The risk equation for predicting an individual’s risk of methotrexate discontinuation with abnormal blood test results by five years follow-up was formulated using the development data. The baseline survival function at t=5 years, a non-parametric estimate of survival function when all predictor values are set to zero, was estimated along with the estimated regression coefficients (β) and the individual’s predictor values (X). This led to the equation for the predicted absolute risk over time^34^:

Predicted risk of methotrexate toxicity associated drug discontinuation at five years=1–S_0_(t_=5_)^exp(βX)^

where S_0_(t_=5_) is the baseline survival function at five years of follow-up and βX is the linear predictor, β_1_x_1_+β_2_x_2_+ . . . +β_p_x_p_.

#### Model internal validation and shrinkage

The performance of the model in terms of calibration (the agreement between predicted and observed risks) was assessed by plotting agreement between predicted and observed outcomes, using a smoothed non-parametric calibration curve across the whole range. A curve close to the 45° line is ideal (quantified by a calibration-in-the-large of zero, calibration slope of 1).

Internal validation was performed to correct model performance estimates for optimism due to overfitting by bootstrapping with replacement 500 samples of the development data. The full model was fitted in each bootstrap sample, and its performance was quantified in the bootstraps (apparent performance) and original samples (test model performance). Model optimism was calculated as the difference between test performance and apparent performance. We estimated a uniform shrinkage factor as the average of calibration slopes from each of the bootstrap models tested in the original sample. This process was repeated for all 10 imputed datasets, and the final uniform shrinkage calculated by averaging across the estimated shrinkage estimates from each imputation. Optimism adjusted estimates of performance for the original model were then calculated as the original apparent performance minus the optimism.

To account for overfitting during the model development process, we multiplied the original β coefficients by the final uniform shrinkage factor and re-estimated the baseline hazards conditional on the shrunken β coefficients to ensure that overall calibration was maintained, producing a final model. We calculated Royston’s D statistic, a measure of discrimination (that is, the ability to discriminate between those with and those without the outcome), interpreted as a log hazard ratio, the exponential of which gives the hazard ratio comparing two groups above or below the median of the linear predictor.^35^ ^36^ We also calculated Royston’s R^2^, a measure of variation explained by the model based on the D statistic, and Harrell’s C statistic, a measure of the model’s predictive accuracy.

#### Model external validation

External validation was performed using data from CPRD Aurum. The study setting, eligibility criteria, outcome, and predictors did not differ from that of the development cohort. We applied the final developed model equation to the validation dataset, and we examined calibration and discrimination as described previously.^35^ ^36^ Calibration of five year risks was examined by plotting agreement between estimated risk from the model and observed outcome risks. In the calibration plot, we divided predicted and observed risks into 10 equally sized groups defined by 10ths of predicted risk. Additionally, we used pseudo-observations to construct smooth calibration curves across these groups through a running non-parametric smoother. Separate graphs were plotted for each imputation of the validation cohort. R^2^ and C statistic were calculated for the validation cohort. Subgroup analyses considered age groups, methotrexate doses, routes of administration, and type of immune mediated inflammatory disease. The supplementary material includes a detailed explanation of the statistical methods used in the study. Stata-MP version 16 was used for all statistical analyses. 

### Patient and public involvement

Patients and members of the public were directly involved in selecting and prioritising the research question. They advised to use readily available datasets for the study rather than conduct an expensive and time consuming clinical trial.

## Results

### Participants

Data for 13 110 and 23 999 participants who contributed 34 298 and 67 150 person years follow-up were included in the development and validation cohorts, respectively (see supplementary figures S1 and S2). Most participants in both cohorts had a diagnosis of rheumatoid arthritis, self-identified as female, and had similar lifestyle factors, comorbidities, and prescriptions (table 1). The median methotrexate dose in both cohorts was 15 mg/week (interquartile range 10-20 mg/week in the development cohort and 12.5-20 mg/week in the validation cohort). Eleven candidate predictors (17 parameters) were included in the model (table 2).

**Table 1:** Distribution of candidate predictors in development (CPRD Gold) and validation (CPRD Aurum) cohorts. Values are number (percentage) unless stated otherwise

Predictors	Development cohort (n=13 110)	Validation cohort (n=23 999)
Mean (SD) age (years)	56.8 (14.8)	57.4 (14.7)
Female sex	8278 (63.1)	15 252 (63.6)
Methotrexate dose:		
Median (interquartile range) dose (mg/week)	15 (10-20)	15 (12.5 20)
Missing data	1053 (8.0)	1537 (6.4)
Body mass index:		
<18.5	198 (1.5)	353 (1.5)
18.5-24.9	3586 (27.4)	6495 (27.1)
25.0-29.9	4402 (33.6)	7712 (32.1)
≥30	4038 (30.8)	6881 (28.7)
Missing data	886 (6.8)	2558 (10.7)
Current smoker:		
No*	10 311 (78.7)	19 555 (81.5)
Yes	2799 (21.4)	4444 (18.5)
Alcohol consumption (units/week):		
Non-drinker	1143 (8.7)	4406 (18.4)
Low (1-14)	6383 (48.7)	10 228 (42.6)
Moderate (15-21)	508 (3.9)	1331 (5.6)
Hazardous (>21)	722 (5.5)	1458 (6.1)
Former drinker	2767 (21.1)	2646 (11.0)
Missing data	1587 (12.1)	3930 (16.4)
Inflammatory conditions:		
Rheumatoid arthritis	8097 (61.8)	15 079 (62.80)
Psoriasis or psoriatic arthritis	3150 (24.0)	5084 (21.2)
Polymyalgia rheumatica or giant cell arteritis	1091 (8.3)	2275 (9.5)
Connective tissue diseases†	296 (2.3)	727 (3.0)
Other seronegative spondyloarthritis‡	476 (3.6)	834 (3.5)
Comorbidities:		
Diabetes mellitus	1700 (13.0)	2689 (11.2)
Chronic kidney disease stage 3	973 (7.4)	2076 (8.7)
Drugs:		
Hydroxychloroquine	1968 (15.0)	4984 (20.8)
5-aminosalicylate or sulfasalazine	2067 (15.8)	3080 (12.8)
Other glucocorticoid sparing drugs§	181 (1.4)	342 (1.4)
Statin	2833 (21.6)	5277 (22.0)
NSAID or high dose aspirin (≥ 300 mg/day)	4888 (37.3)	7471 (31.1)
Paracetamol (acetaminophen)	2173 (16.6)	3620 (15.1)
Proton pump inhibitor	5870 (44.8)	10 687 (44.5)
Antiepileptic¶	118 (0.9)	239 (1.0)
Blood test abnormalities:		
Cytopenia or raised liver enzyme levels within first six months of first primary care methotrexate prescription	2512 (19.2)	4429 (18.5)
Median (interquartile range) follow-up (years)	2.35 (0.91-4.83)	2.73 (1.05-5.00)
Outcome events	854 (6.5)	1486 (6.2)

CPRD=Clinical Practice Research Datalink; NSAID=non-steroidal anti-inflammatory drug.

One unit of alcohol equals 10 mL or 8 g of pure alcohol.

* Includes non-smokers, former smokers, and smoking status not available.

† Includes lupus, systemic sclerosis, myositis, and small vessel vasculitis.

‡ Includes ankylosing spondylitis, reactive arthritis, and inflammatory bowel disease associated inflammatory arthritis.

§ Includes leflunomide, azathioprine, 6-mercaptopurine, ciclosporin, tacrolimus, and mycophenolate mofetil.

¶ Includes carbamazepine, levetiracetam, and valproate.

**Table 2:** Final model hazard ratios and β coefficients before shrinkage

Predictors	Adjusted hazard ratio (95% CI)*	β coefficient
Age (years)	1.00 (1.00 to 1.01)	−0.0005886
Female sex	0.96 (0.83 to1.11)	−0.0412595
Methotrexate dose (mg/day)	1.01 (0.93 to 1.09)	0.0111752
Body mass index	0.99 (0.98 to 1.00)	−0.0100983
Smoking status:		
Non-smoker, NR, former smoker	Reference	-
Current smoker	1.06 (0.89 to 1.25)	0.0545787
Alcohol consumption (units/week):		
Non-drinker	Reference	-
Low (1-14)	0.93 (0.73 to 1.17)	−0.0745235
Moderate (15-21)	0.79 (0.52 to 1.19)	−0.2376144
Hazardous (>21)	1.11 (0.79 to 1.56)	0.1062592
Former drinker	0.95 (0.74 to 1.22)	−0.0555338
Inflammatory conditions:		
Rheumatoid arthritis	Reference	-
Psoriasis or psoriatic arthritis	1.14 (0.96 to 1.36)	0.1339634
Polymyalgia rheumatica or giant cell arteritis	0.99 (0.75 to 1.31)	−0.0111359
Connective tissue diseases*	1.34 (0.87 to 2.07)	0.2919058
Other seronegative spondyloarthritis†	1.30 (0.91 to 1.87)	0.2636568
Comorbidities:		
Diabetes mellitus	1.25 (1.02 to 1.53)	0.2225364
Chronic kidney disease stage 3	2.01 (1.63 to 2.49)	0.7005654
Drugs:		
Hydroxychloroquine	0.90 (0.72 to 1.11)	−0.1073611
5-aminosalicylate or sulfasalazine	0.92 (0.76 to 1.12)	−0.0791611
Other glucocorticoid sparing drugs‡	1.11 (0.66 to 1.85)	0.1000699
Statin	1.11 (0.93 to 1.32)	0.1021201
NSAID or high dose aspirin (≥300 mg/day)	0.97 (0.83 to 1.12)	−0.0334514
Paracetamol (acetaminophen)	1.17 (0.98 to 1.40)	0.1608563
Proton pump inhibitor	1.02 (0.88 to 1.18)	0.0170423
Antiepileptic§	1.68 (0.92 to 3.05)	0.5164347
Blood test abnormalities:		
Cytopenia or raised liver enzyme levels within first six months of first primary care methotrexate prescription	2.97 (2.57 to 3.41)	1.086389

NR=not recorded; NSAID=non-steroidal anti-inflammatory drug.

One unit of alcohol equals 10 mL or 8 g of pure alcohol.

* Includes lupus, systemic sclerosis, myositis, and small vessel vasculitis.

† Includes ankylosing spondylitis, reactive arthritis, and inflammatory bowel disease associated inflammatory arthritis.

‡ Includes leflunomide, azathioprine, 6-mercaptopurine, ciclosporin, tacrolimus, and mycophenolate mofetil.

§ Includes carbamazepine, levetiracetam, and valproate.

### Model development

In the development dataset, 854 outcomes occurred in 6.5% of patients (n=13 110) during the follow-up period, at a rate of 24.90 (95% confidence interval 23.28 to 26.63) per 1000 person years. Of the 854 outcomes, 352 (41.2%) were due to cytopenia, 293 (34.3%) were due to raised liver enzyme levels, and 209 (24.5%) were due to worsening renal function. Diabetes, chronic kidney disease stage 3, and either cytopenia or raised liver enzyme levels during the first six months of a methotrexate prescription were strong predictors of drug discontinuation, with before shrinkage adjusted hazard ratios of 1.25 (95% confidence interval 1.02 to 1.53), 2.01 (1.63 to 2.49), and 2.97 (2.57 to 3.41), respectively (table 2).

**Fig 1:**
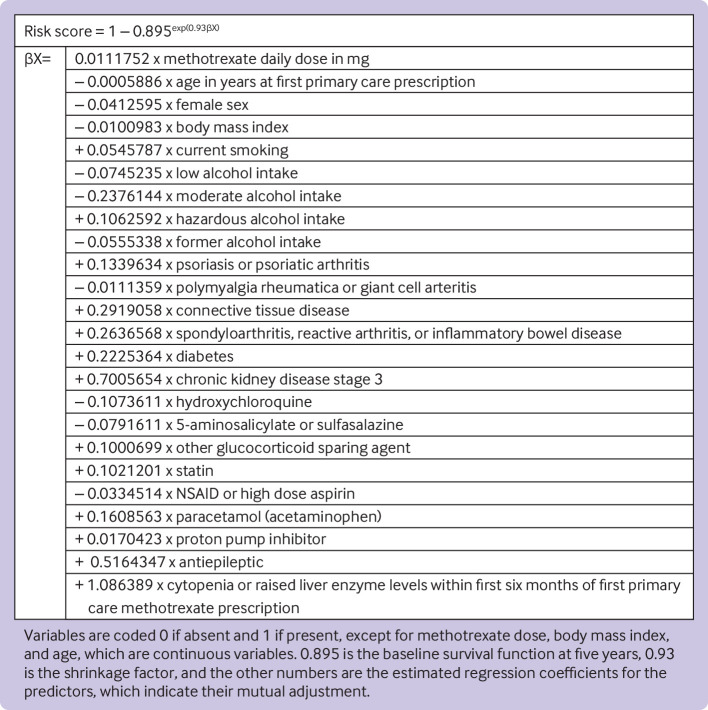
Equation to predict risk of methotrexate discontinuation owing to abnormal monitoring blood test results after six months of primary care prescription and within next five years. Connective tissue diseases include lupus, systemic sclerosis, myositis, and small vessel vasculitis. Other immunosuppressant glucocorticoid sparing agents include leflunomide, azathioprine, ciclosporin, and tacrolimus. Antiepileptics include carbamazepine, levetiracetam, and sodium valproate. Blood test abnormality defined as either cytopenia (neutrophil count <2×10^9^/L, total leucocyte count <4×10^9^/L, or platelet count <150×10^9^/L) or raised transaminase levels (alanine transaminase and/or aspartate transaminase >35 IU/L) during the first six months of a prescription for methotrexate in primary care

Before shrinkage, the calibration slope in the development data was 1.00 (95% confidence interval 0.89 to 1.11). From the bootstrap, a uniform shrinkage factor of 0.93 was obtained and used to shrink predictor coefficients in the final model for optimism, and after re-estimation the final model’s cumulative baseline survival function (S_0_) was 0.895 at five years of follow-up (fig 1, also see supplementary material).

### Model performance in development cohort

**Fig 2:**
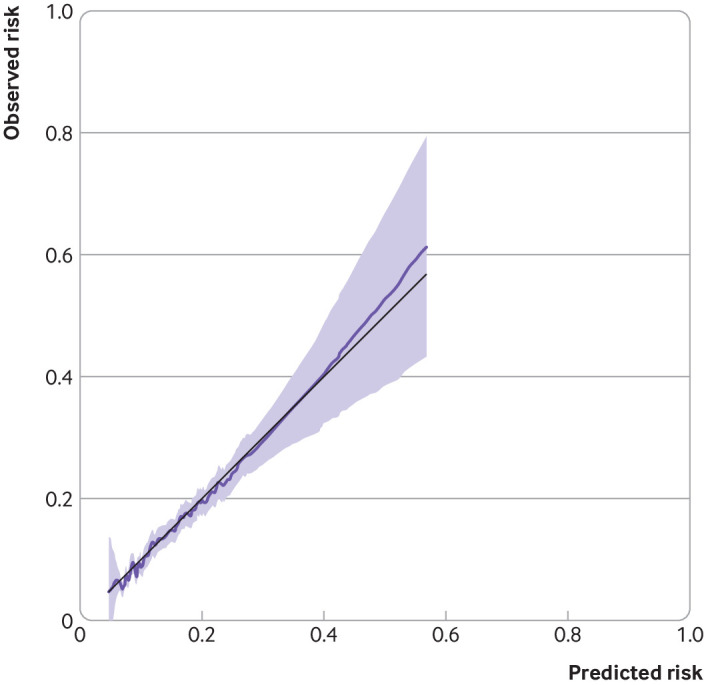
Calibration of a prognostic model for methotrexate discontinuation with abnormal monitoring blood test results at five years in development cohort. Data from a single imputed dataset were used for illustration. Baseline survival function (S_0_) was 0.895 at five years of follow-up. Black line reflects perfect prediction

Supplementary figure S3 shows a calibration plot of the final (ie, after shrinkage) model at five years, with average model predictions closely matching the average observed outcome probabilities across all 10 groups of patients, with confidence intervals overlapping the 45° line (perfect prediction line). As most patients had a low risk of the outcome (see supplementary figure S4), most of the groups are clustered at the bottom left of the calibration plot. Figure 2 shows the smoothed calibration curve at five years. The Royston D statistic was 0.89 (95% confidence interval 0.78 to 1.00), corresponding to a hazard ratio of 2.44 (95% confidence interval 2.18 to 2.72) when comparing the risk groups above and below the median of linear predictor. The optimism adjusted Royston D statistic was 0.79, corresponding to a hazard ratio of 2.20, obtained by exponentiating the D statistic. The optimism corrected R^2^ was 0.13 (table 3).

### Model performance in validation cohort

**Fig 3:**
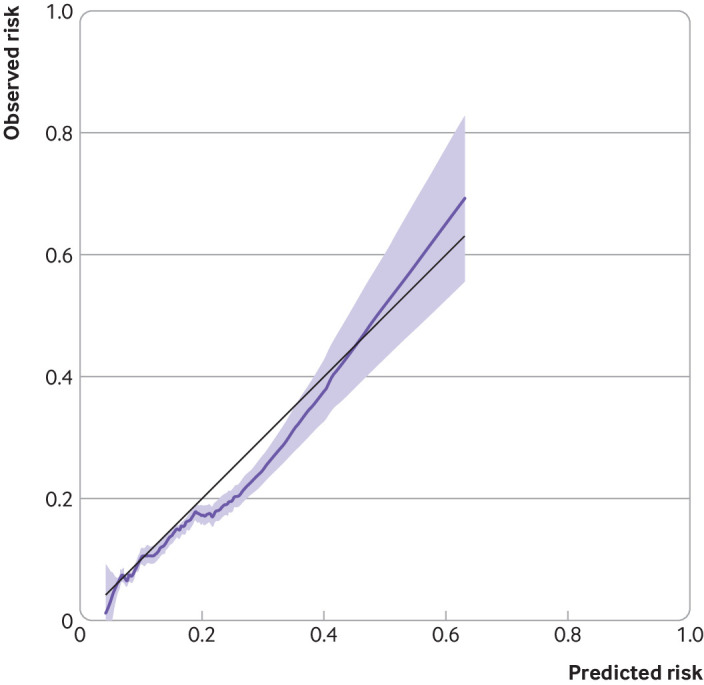
Calibration of a prognostic model for methotrexate discontinuation with abnormal monitoring blood test results at five years in validation cohort. Data from a single imputed dataset were used for illustration. Baseline survival function (S_0_) was 0.895 at five years of follow-up. Black line reflects perfect prediction

Overall, 1486 outcomes occurred in 6.2% patients (n=23 999) at a rate of 22.13 (95% confidence interval 21.03 to 23.28) per 1000 person years in the validation cohort. The calibration slope across the five year follow-up period was 0.94 (95% confidence interval 0.85 to 1.02). The calibration plot showed reasonable correspondence between observed and predicted risk at five years across the groups defined by 10ths of risk (see supplementary figure S5). Most groups clustered at the bottom left of the calibration plot owing to low risk of outcome for most patients (supplementary figure S6). The smoothed calibration curve also showed good agreement of the predicted risk to the observed risk (fig 3). Model performance was also tested at years 1, 2, 3, and 4 (see supplementary figures S7-S10), and a similar pattern was observed.

Model discrimination in the development and validation data was broadly similar (table 3). The Royston D statistic in the validation cohort was 0.75 (95% confidence interval 0.67 to 0.83), corresponding to a hazard ratio of 2.12 (95% confidence interval 1.95 to 2.29). The R^2^ and Harrell’s C statistic were 0.12 (95% confidence interval 0.10 to 0.14) and 0.64 (95% confidence interval 0.62 to 0.65), respectively. The model calibration slope and discrimination were similar across subgroups defined by age (<60 years or ≥60 years), methotrexate dose (≤15 mg/week or >15 mg/week), route of administration (oral or subcutaneous), and type of immune mediated inflammatory disease (rheumatoid arthritis or others) (see supplementary table S1 and figures S11-S14).

**Table 3:** Model diagnostics

Measure	Apparent performance (95% CI)*	Test performance (95% CI)†	Average optimism‡	Optimism corrected performance§	Performance in external validation (CPRD Aurum)
Overall calibration slope	1.00 (0.89 to 1.11)	0.93 (0.83 to 1.03)	0.07	0.93	0.94 (0.85 to 1.02)
Royston D statistic	0.89 (0.78 to 1.00)	0.85 (0.73 to 0.96)	0.10	0.79	0.75 (0.67 to 0.83)
R^2^	0.16 (0.13 to 0.19)	0.15 (0.11 to 0.19)	0.03	0.13	0.12 (0.10 to 0.14)
Harrell’s *C*	0.66 (0.64 to 0.68)	0.65 (0.64 to 0.66)	0.01	0.65	0.64 (0.62 to 0.65)

CPRD=Clinical Practice Research Datalink.

* Estimated directly from data that was used to develop the model.

† Determined by executing full model in each bootstrap sample of the development dataset (500 samples with replacement), calculating bootstrap performance, and applying same model in original sample.

‡ Average difference between model performance in bootstrap sample of the development dataset and performance in the development dataset.

§ Obtained by subtracting average optimism from apparent performance.

### Worked examples

Ten anonymised patient profiles, one from the middle of each of the 10 groups defined by 10ths of predicted risk were selected from the development cohort, the higher the decile group the higher the risk, and the risk equation was applied to each (see supplementary table S2). The cumulative probability of outcome over five years ranged from 7.1% in the middle of the first group to 9.4% in the middle of the seventh group, and 23.7% in the middle of the 10th group.

## Discussion

We developed and externally validated a prognostic model for predicting the likelihood of methotrexate discontinuation owing to abnormal blood test results that utilises disease and demographic factors ascertained during routine clinic visits in either primary or secondary care. Our prognostic model performed well in predicting outcomes by five years and did so in clinically relevant subgroups defined by age group, type of immune mediated inflammatory disease, methotrexate dose, and route of administration. Strong independent predictors were cytopenia or raised liver enzyme levels, or both, in the six months before start of follow-up, chronic kidney disease stage 3, and diabetes. The last two associations could be related to reduced methotrexate clearance in patients with chronic kidney disease and to drug induced liver injury and non-alcoholic fatty liver disease in people with diabetes.^37^

### Strengths and limitations of this study

Strengths of this study included the use of a large, real world, and nationally representative dataset for model development, and a similar independent dataset for external validation. The study population included patients with a range of immune mediated inflammatory diseases, and therefore the results have broad generalisability. Expert members of a multidisciplinary team selected the prognostic factors based on their scientific knowledge and clinical experience. Outcome definition required the abnormal blood test result to be associated with methotrexate discontinuation, thus allowing the model to predict clinically relevant outcomes. Finally, information on factors included in this prognostic model are easily ascertainable during routine care, making the model simple to use and enabling it to be incorporated into electronic health records.

Some limitations of this study need consideration. Firstly, we did not have access to the date when patients were first prescribed methotrexate in the hospital clinic. We did, however, have accurate information on the date of first methotrexate prescription in primary care, and therefore our model is fit to risk stratify monitoring from six months after a first prescription in primary care. We also did not have data on the concurrent use of biologics, as these are prescribed in hospital. Blood test abnormalities during long term treatment with biologics are uncommon, and the British Society for Rheumatology monitoring guidelines show no evidence to suggest that concurrently prescribed biologics increase methotrexate toxicity.^38^ We did not have information on disease activity (ie, how well the disease was controlled) as these data are not recorded in CPRD. Increased disease activity is not expected to cause abnormal monitoring blood test results directly but may result in the use of higher doses of methotrexate or combination glucocorticoid sparing treatment, both of which we included in the model. It is possible that abnormal blood test results are due to another disorder and not to methotrexate. In our previous validation study, only 5.4% of abnormal blood test results could be explained by another disorder.^17^ Although the external validation dataset was distinct from the model development dataset, it also originated from UK general practice. We recommend that our model be validated in a dataset from another country. Our imputation strategy is compatible with all predictors being available at implementation, and further research would need to consider how to handle missing predictor values at that point. Moreover, only 1.7% (n=224/13 110) and 1.6% (n=387/23 999) of patients in the development and validation cohorts, respectively, had a predicted risk >30% resulting in uncertainty about predictions in those at very high risk. We did not perform competing risk regression to account for the competing risk of death, although the proportions of deaths were small (0.1%) in the development cohort (n=12/13 110) and validation cohort (n=30/23 999).

In terms of external validity, most of the patients in the cohort had rheumatoid arthritis, thereby potentially limiting generalisability of these findings to other diseases. The model performance was, however, comparable when we considered patients with rheumatoid arthritis or other immune mediated inflammatory diseases separately. We did not use methotrexate dose reduction to define outcomes because of missing data on doses between some prescriptions. Despite such events not being considered, the study had sufficient power for the parameters included. We excluded patients with a previous diagnosis of serious liver, haematological, or renal diseases as these conditions often contraindicate methotrexate use and may additionally cause uncertainty with outcome ascertainment. On the basis of this requirement, we excluded only 1.5% of patients (n=1060/69 154) considered for inclusion in the development and validation cohorts. In the prognostic model, we considered milder liver, haematological, or renal diseases in which low dose weekly methotrexate is often prescribed. Finally, the model is derived from a primary care dataset and may not be applicable to patients with complex comorbidities prescribed and monitored exclusively in secondary care. This is, however, unlikely to be an important limitation because such patients are uncommon owing to the availability of other treatments.

Overall, 9% of the development cohort (n=1186) had ≥90 days gap in methotrexate prescriptions without any blood test results available in CPRD within 60 days of the last prescription date. We treated these as random censoring events not associated with an outcome. Not all of these gaps in treatment would be related to abnormal blood test results. Other reasons may include lack of efficacy, disease remission, other side effects, and patient choice (eg, to start a family). Nevertheless, it is possible that some patients who actually experienced an outcome were misclassified owing to missing data. This would potentially worsen model performance. We are reassured this problem is not major as it concerned a small proportion of people, the model calibration was close to 1, and discrimination was high.

### Policy implications

The risk score output from the prognostic model may be used to decide individual monitoring strategies after the first six months of a prescription for methotrexate in primary care—that is, patients at low risk of toxicity could be advised to undergo less frequent monitoring blood tests, whereas those at high risk of toxicity undergo more frequent testing. Such decision making requires shared input, taking into account individualised risk scores, patient preferences, recommendations of health professionals, and updated guidelines. It is beyond the scope of this study to recommend specific risk thresholds at which current clinical practice may be changed.

The frequency of monitoring blood tests is decided according to recommendations of specialist societies such as the British Society of Rheumatology and British Association of Dermatology, and these thresholds are best decided by guideline writing groups that are external to the research team and include broader clinical and patient representation.

Other prognostic models are widely used where the risk of future events is calculated as a continuous score and the threshold at which clinical practice is changed is recommended in specialist society guidelines. For example, the fracture risk algorithm (FRAX) calculates the risk of future osteoporotic fractures as a continuous score, and the recommended thresholds for lifestyle interventions, bone density measurement, or drug treatments are decided according to guidance from the National Osteoporosis Guideline Group.

Within these limitations, it would seem reasonable to offer patients at relatively low risk (eg, <10% over five years, representing 68.4% of the validation cohort) six monthly or annual testing, whereas those with moderate risk (eg, 10-20% over five years, representing 20.9% of the validation cohort) might continue with the current testing every three months, and those with high risk (eg, >20% over five years, representing 10.7% of the validation cohort) undergo more frequent testing.

Lengthening the time between monitoring blood tests would save patients’ and health professionals’ time, minimise discomfort from unnecessary venepunctures, and conserve healthcare resources, because patients with well controlled immune mediated inflammatory diseases undergo quarterly monitoring blood tests at their GP’s surgery or at hospital or community phlebotomy services and are only followed-up in specialist clinics annually. Patients taking methotrexate are usually adherent to monitoring recommendations,^12^ and our model, if implemented, should reduce the volume of monitoring blood tests. It is important that the results of this study are not used to risk stratify monitoring in patients who have recently started methotrexate treatment because we did not use data on such patients in our study. In the UK, it typically takes six months to stabilise methotrexate doses in patients before prescribing and monitoring can be handed over to GPs. In healthcare systems where specialists continue to prescribe and monitor methotrexate indefinitely, this model may be used to risk stratify monitoring from one year after the first methotrexate prescription. The results of this study need to be considered by guideline writing groups to develop new recommendations for blood test monitoring.

### Conclusion

We have developed and externally validated a prognostic model for predicting the likelihood of methotrexate discontinuation owing to abnormal blood test results that can be readily used in clinical practice. Future research should evaluate the cost effectiveness and patients’ and health professionals’ acceptability of a risk stratified monitoring strategy. Further validation studies should be performed in other populations from outside the UK and should consider fewer predictor parameters.

**What is already known on this topic**

Clinically significant cytopenia, raised liver enzyme levels, and acute kidney injury that require drug discontinuation are infrequent after the first year of methotrexate treatment
Despite a lack of evidence, all patients established on low dose weekly methotrexate (≤25 mg) are required to undergo blood tests every three months indefinitely for early detection of toxicity
It is not known whether methotrexate related toxicities can be predicted and whether monitoring could be risk stratified

**What this study adds**

A prognostic model was developed and validated that discriminated patients at varying risk of methotrexate toxicity during long term treatment
The model performed well across age groups, inflammatory conditions, methotrexate doses, and routes of administration
The findings are useful for updating guidelines on blood test monitoring during long term methotrexate treatment for immune mediated inflammatory diseases

## Data Availability

The study protocol is available from www.cprd.com.

## Web extra

Extra material supplied by authors

Supplementary information: Additional methods, tables 1 and 2, and figures S1-S14

Supplementary information: Excel spreadsheet showing risk calculator

## Notes

Contributors: GN, MJG, HCW, TC, MWT, GPA, CPF, CDM, DAW, MDS, RDR, and AA designed the study. GN analysed the data supervised by MJG, TC, RDR, and AA. GN, MJG, HCW, TC, MWT, GPA, CPF, CDM, DAW, MDS, RDR, and AA interpreted the data. AA and GN drafted the manuscript. AA led the revision of the manuscript. All authors critically evaluated and revised the manuscript. AA is the guarantor. The corresponding author attests that all listed authors meet authorship criteria and that no others meeting the criteria have been omitted.

The lead author (AA) affirms that the manuscript is an honest, accurate, and transparent account of the study being reported; that no important aspects of the study have been omitted; and that any discrepancies from the study as planned (and, if relevant, registered) have been explained.

Dissemination to participants and related patient and public communities: The results of this study will be disseminated to the guideline writing groups of the British Society of Rheumatology, British Association of Dermatologists, and British Gastroenterology Society. Results will also be sent to Royal College of General Practitioners. An infographic and a video explaining the study results will be co-produced with patient and public involvement input and disseminated to patients and the public.

Provenance and peer review: Not commissioned; externally peer reviewed.

## References

[1] Gossec L Baraliakos X Kerschbaumer A . EULAR recommendations for the management of psoriatic arthritis with pharmacological therapies: 2019 update. Ann Rheum Dis 2020;79:700-12. 10.1136/annrheumdis-2020-217159 32434812 PMC7286048

[2] Smolen JS Landewé RBM Bijlsma JWJ . EULAR recommendations for the management of rheumatoid arthritis with synthetic and biological disease-modifying antirheumatic drugs: 2019 update. Ann Rheum Dis 2020;79:685-99. 10.1136/annrheumdis-2019-216655 31969328

[3] Dejaco C Singh YP Perel P European League Against Rheumatism American College of Rheumatology . 2015 Recommendations for the management of polymyalgia rheumatica: a European League Against Rheumatism/American College of Rheumatology collaborative initiative. Ann Rheum Dis 2015;74:1799-807. 10.1136/annrheumdis-2015-207492 26359488

[4] Fraenkel L Bathon JM England BR . 2021 American College of Rheumatology guideline for the treatment of rheumatoid arthritis. Arthritis Rheumatol 2021;73:1108-23. 10.1002/art.41752 34101376

[5] Menter A Gelfand JM Connor C . Joint American Academy of Dermatology-National Psoriasis Foundation guidelines of care for the management of psoriasis with systemic nonbiologic therapies. J Am Acad Dermatol 2020;82:1445-86. 10.1016/j.jaad.2020.02.044 32119894

[6] Donahue KE Schulman ER Gartlehner G . Comparative Effectiveness of Combining MTX with Biologic Drug Therapy Versus Either MTX or Biologics Alone for Early Rheumatoid Arthritis in Adults: a Systematic Review and Network Meta-analysis. J Gen Intern Med 2019;34:2232-45. 10.1007/s11606-019-05230-0 31388915 PMC6816735

[7] Xie Y Liu Y Liu Y . Are biologics combined with methotrexate better than biologics monotherapy in psoriasis and psoriatic arthritis: A meta-analysis of randomized controlled trials. Dermatol Ther 2021;34:e14926. 10.1111/dth.14926 33655646

[8] Kennedy NA Heap GA Green HD UK Inflammatory Bowel Disease Pharmacogenetics Study Group . Predictors of anti-TNF treatment failure in anti-TNF-naive patients with active luminal Crohn’s disease: a prospective, multicentre, cohort study. Lancet Gastroenterol Hepatol 2019;4:341-53. 10.1016/S2468-1253(19)30012-3 30824404

[9] Edwards CJ Campbell J van Staa T Arden NK . Regional and temporal variation in the treatment of rheumatoid arthritis across the UK: a descriptive register-based cohort study. BMJ Open 2012;2:e001603. 10.1136/bmjopen-2012-001603 23144258 PMC3533005

[10] Steffen A Holstiege J Klimke K Akmatov MK Bätzing J . Patterns of the initiation of disease-modifying antirheumatic drugs in incident rheumatoid arthritis: a German perspective based on nationwide ambulatory drug prescription data. Rheumatol Int 2018;38:2111-20. 10.1007/s00296-018-4161-7 30306254 PMC6208685

[11] Egeberg A Skov L Gislason GH Thyssen JP Mallbris L . Incidence and Prevalence of Psoriasis in Denmark. Acta Derm Venereol 2017;97:808-12. 10.2340/00015555-2672 28417141

[12] Fraser SD Lin SX Stammers M . Persistently normal blood tests in patients taking methotrexate for RA or azathioprine for IBD: a retrospective cohort study. Br J Gen Pract 2022;72:e528-37. 10.3399/BJGP.2021.0595 35256384 PMC8936183

[13] Tugwell P Bennett K Gent M . Methotrexate in rheumatoid arthritis. Indications, contraindications, efficacy, and safety. Ann Intern Med 1987;107:358-66. 10.7326/0003-4819-107-2-358 3304050

[14] Weinblatt ME Coblyn JS Fox DA . Efficacy of low-dose methotrexate in rheumatoid arthritis. N Engl J Med 1985;312:818-22. 10.1056/NEJM198503283121303 3883172

[15] Ledingham J Gullick N Irving K BSR and BHPR Standards, Guidelines and Audit Working Group . BSR and BHPR guideline for the prescription and monitoring of non-biologic disease-modifying anti-rheumatic drugs. Rheumatology (Oxford) 2017;56:865-8. 10.1093/rheumatology/kew479 28339817

[16] Warren RB Weatherhead SC Smith CH . British Association of Dermatologists’ guidelines for the safe and effective prescribing of methotrexate for skin disease 2016. Br J Dermatol 2016;175:23-44. 10.1111/bjd.14816 27484275

[17] Nakafero G Grainge MJ Card T . What is the incidence of methotrexate or leflunomide discontinuation related to cytopenia, liver enzyme elevation or kidney function decline? Rheumatology (Oxford) 2021;60:5785-94. 10.1093/rheumatology/keab254 33725120 PMC8645271

[18] Herrett E Gallagher AM Bhaskaran K . Data Resource Profile: Clinical Practice Research Datalink (CPRD). Int J Epidemiol 2015;44:827-36. 10.1093/ije/dyv098 26050254 PMC4521131

[19] Wolf A Dedman D Campbell J . Data resource profile: Clinical Practice Research Datalink (CPRD) Aurum. Int J Epidemiol 2019;48:1740-1740g. 10.1093/ije/dyz034 30859197 PMC6929522

[20] Moons KG Altman DG Reitsma JB . Transparent Reporting of a multivariable prediction model for Individual Prognosis or Diagnosis (TRIPOD): explanation and elaboration. Ann Intern Med 2015;162:W1-W73. 10.7326/M14-0698 25560730

[21] Lewis JD Bilker WB Weinstein RB Strom BL . The relationship between time since registration and measured incidence rates in the General Practice Research Database. Pharmacoepidemiol Drug Saf 2005;14:443-51. 10.1002/pds.1115 15898131

[22] Abhishek A Doherty M Kuo CF Mallen CD Zhang W Grainge MJ . Rheumatoid arthritis is getting less frequent-results of a nationwide population-based cohort study. Rheumatology (Oxford) 2017;56:736-44. 10.1093/rheumatology/kew468 28064207 PMC5850292

[23] Jones S. Methotrexate (oral or subcutaneous) for psoriasis and other dermatological conditions, 2018. https://mm.wirral.nhs.uk/document_uploads/shared-care/Methotrexate_for_derm_diseases_shared_care_v2.pdf.

[24] NHS England. National shared care protocol: Methotrexate (oral and subcutaneous) for patients in adult services (excluding cancer care), 2022 https://www.england.nhs.uk/wp-content/uploads/2022/07/B1621_xvii_methotrexate-oral-and-subcutaneous-for-patients-in-adult-services-excluding-cancer-care.docx.

[25] Kidney Disease: Improving Global Outcomes (KDIGO) Acute Kidney Injury Work Group. KDIGO Clinical Practice Guideline for Acute Kidney Injury. *Kidney Inter Suppl* 2012:2:1-138.

[26] Chalasani N Björnsson E . Risk factors for idiosyncratic drug-induced liver injury. Gastroenterology 2010;138:2246-59. 10.1053/j.gastro.2010.04.001 20394749 PMC3157241

[27] Safy-Khan M de Hair MJH Welsing PMJ van Laar JM Jacobs JWG Society for Rheumatology Research Utrecht (SRU) . Current Smoking Negatively Affects the Response to Methotrexate in Rheumatoid Arthritis in a Dose-responsive Way, Independently of Concomitant Prednisone Use. J Rheumatol 2021;48:1504-7. 10.3899/jrheum.200213 33526623

[28] Bressolle F Bologna C Kinowski JM Sany J Combe B . Effects of moderate renal insufficiency on pharmacokinetics of methotrexate in rheumatoid arthritis patients. Ann Rheum Dis 1998;57:110-3. 10.1136/ard.57.2.110 9613341 PMC1752537

[29] Carmichael SJ Beal J Day RO Tett SE . Combination therapy with methotrexate and hydroxychloroquine for rheumatoid arthritis increases exposure to methotrexate. J Rheumatol 2002;29:2077-83. 12375315

[30] Meijer B Wilhelm AJ Mulder CJJ Bouma G van Bodegraven AA de Boer NKH . Pharmacology of Thiopurine Therapy in Inflammatory Bowel Disease and Complete Blood Cell Count Outcomes: A 5-Year Database Study. Ther Drug Monit 2017;39:399-405. 10.1097/FTD.0000000000000414 28489727 PMC5538301

[31] Dirven L Klarenbeek NB van den Broek M . Risk of alanine transferase (ALT) elevation in patients with rheumatoid arthritis treated with methotrexate in a DAS-steered strategy. Clin Rheumatol 2013;32:585-90. 10.1007/s10067-012-2136-8 23224330

[32] Riley RD Ensor J Snell KIE . Calculating the sample size required for developing a clinical prediction model. BMJ 2020;368:m441. 10.1136/bmj.m441 32188600

[33] Schafer JL . Multiple imputation: a primer. Stat Methods Med Res 1999;8:3-15. 10.1177/096228029900800102 10347857

[34] Steyerberg EW . Clinical Prediction Models: A Practical Approach to Development, Validation, and Updating. Springer International Publishing, 2019 10.1007/978-3-030-16399-0.

[35] Royston P Altman DG . External validation of a Cox prognostic model: principles and methods. BMC Med Res Methodol 2013;13:33. 10.1186/1471-2288-13-33 23496923 PMC3667097

[36] Cox DR . Note on Grouping. J Am Stat Assoc 1957;52:543-7 10.1080/01621459.1957.10501411.

[37] Mori S Arima N Ito M Fujiyama S Kamo Y Ueki Y . Non-alcoholic steatohepatitis-like pattern in liver biopsy of rheumatoid arthritis patients with persistent transaminitis during low-dose methotrexate treatment. PLoS One 2018;13:e0203084. 10.1371/journal.pone.0203084 30142184 PMC6108522

[38] Holroyd CR Seth R Bukhari M . The British Society for Rheumatology biologic DMARD safety guidelines in inflammatory arthritis. Rheumatology (Oxford) 2019;58:e3-42. 10.1093/rheumatology/key208 30137552

