# Supplementary material for: Risk stratified monitoring for methotrexate toxicity in immune mediated inflammatory diseases: prognostic model development and validation using primary care data from the UK
Source: BMJ. 2023 May 30;381:e074678. doi: 10.1136/bmj-2022-074678 (PMC10227711; doi:10.1136/bmj-2022-074678)
Supplement: Supplementary file 1 — Supplementary information: Additional methods, tables 1 and 2, and figures S1-S14 [file nakg074678.ww1.pdf]

Contents

Supplementary methods: an explanation of prognostic model development and validation. .. 2

Supplementary Table 1: Model diagnostics: Subgroup performance indicators in external validation dataset (CPRD Aurum). 4

Supplementary Table 2: Patient characteristics at the midpoint of each decile of predicted risk from a randomly selected imputation dataset.... 5

Figure S1: Model development cohort: Study population selection 6

Figure S2: Model validation cohort: Study population selection 7

Figure S3: Calibration of a prognostic model for methotrexate discontinuation with abnormal monitoring blood-test results at 5 years in the derivation cohort 8

Figure S4: Distribution of predicted risk of discontinuation with abnormal monitoring blood-test results in the derivation cohort at 5 years 9

Figure S5: Calibration of a prognostic model for methotrexate discontinuation with abnormal monitoring blood-test results at 5 years in the validation cohort.... 10

Figure S6: Distribution of predicted risk of discontinuation with abnormal monitoring blood-test results in the validation cohort at 5 years.... 11

.... 11

Figure S7: Calibration of a prognostic model for methotrexate discontinuation with abnormal monitoring blood-test results at 1 year in the validation cohort.... 12

Figure S8: Calibration of a prognostic model for methotrexate discontinuation with abnormal monitoring blood-test results at 2 years in the validation cohort.... 13

Figure S9: Calibration of a prognostic model for methotrexate discontinuation with abnormal monitoring blood-test results at 3 years in the validation cohort.... 14

Figure S10: Calibration of a prognostic model for methotrexate discontinuation with abnormal monitoring blood-test results at 4 years in the validation cohort 15

Figure S11: Calibration of a prognostic model for methotrexate discontinuation with abnormal monitoring blood-test results at 5 years in the validation cohort: stratified according to age 16

Figure S12: Calibration of a prognostic model for methotrexate discontinuation with abnormal monitoring blood-test results at 5 years in the validation cohort: stratified according to inflammatory disease type.... 17

Figure S13: Calibration of a prognostic model for methotrexate discontinuation with abnormal monitoring blood-test results at 5 years in the validation cohort: stratified according to route of administration. 18

Figure S14: Calibration of a prognostic model for methotrexate discontinuation with abnormal monitoring blood-test results at 5 years in the validation cohort: stratified according to methotrexate dose.... 19

Supplementary methods: list of conditions excluded from model development cohort. 20

Supplementary methods: list of conditions excluded from model validation cohort. 28

Supplementary methods: an explanation of prognostic model development and validation.

In a time to event analysis, a predicted probability of survival is calculated for each person in a study. In our study we selected survival at 5 years. The predicted probability is calculated from the baseline survival function at a specified time (the same for everyone) and a prediction score which is based on the weight of the predictor (β coefficient) and the value each person takes for that predictor variable (different for everyone). Importantly, in the case of a Cox regression model, beta coefficients are constant throughout the study period so choice of different survival times will not affect the relative ordering of people in the study when ranked according to their predicted survival. Predicted event risk (or probability) is one minus predicted survival.

Discrimination represents the ability of a model to distinguish people who developed the event of interest (discontinuation of methotrexate) from those who did not. We used two measures of discrimination in this paper. The Royston D-statistic represents the comparison of survival curves between people with predicted risks lower and higher than the median predicted risk. The D-statistic is interpreted as the log hazard ratio (interpreted in the same way as an HR for any other variable). The R2D values presented in the paper are based on this D-statistic. It is an estimate of the proportion of variance in the outcome which can be explained by the prediction score. The second measure we included in response to a reviewer comment was Harrell's C-statistic. This represents a concordance probability whereby if two people are randomly selected from the data, it is the probability the one with the shortest survival time has the higher predicted risk.

Calibration estimates how well the predicted values from the model agree with the observed risk. The calibration slope is the regression coefficient obtained when predicted probabilities are plotted against the observed outcomes. The ideal value for the calibration slope is 1. A value much less than 1 indicates overfitting, with predictions too high for high risk patients and too low for low risk patients. In contrast, a value higher than 1 indicates that risk estimates are too moderate at the extremes. These values are obtained via fitting a smoothed calibration curve, meaning we do not need to manually define risk groups. We also assessed calibration by defining 10 equal sized groups and plotted observed risk for each group (difference between Figure S3a and S3b in the paper)

Shrinkage is an important stage of developing a prognostic model to take account of overfitting. It is based on the principle that when a model is tested with new data we would expect it to perform less well than in the data used to develop the model. Therefore, the beta coefficients derived when we build a prognostic model in the derivation cohort often over-estimate the degree of risk separation. This means the model may underperform in a new dataset if the 'true' beta coefficients were lower than those we estimated. This bias is corrected by repeating the modelling process in 500 samples drawn with replacement from the original sample and comparing model performance between the original and bootstrapped samples, which gives us

an idea of how well our model may perform in new data whilst still using our original data (why it is called internal validation). From this we estimate a uniform shrinkage factor (value below 1), which is applied to all model coefficients in order to obtain our final model. Finally, we considered the use of fractional polynomials for the continuous predictor variables. This was to account for the fact that for variables such as age the relationship between predictor and outcome may not be linear, i.e. the increase in risk from 50 to 60 years is not the same as from 80 to 90 years, in which case treating continuous variables as linear will result in a more poorly performing model. Transformed variables did not perform any better than treating the variables as linear in predicting the outcomes, so it was found not to be necessary to transform the variables in this instance.

Supplementary Table 1: Model diagnostics: Subgroup performance indicators in external validation dataset (CPRD Aurum).

Prognostic factors and subgroups	Number (n)	Overall calibration slope (95% CI)	Royston D statistic (95% CI)
Age category			
< 60 years	12,843	0.96 (0.83 to 1.09)	0.69 (0.57 to 0.81)
≥ 60 years	11,156	0.91 (0.79 to 1.03)	0.77 (0.66 to 0.88)
Methotrexate dose			
≤15 mg/week	14,867	0.91 (0.79 to 1.02)	0.71 (0.61 to 0.81)
>15 mg/week	9,132	0.98 (0.84 to 1.13)	0.81 (0.68 to 0.94)
Route of administration			
Oral	23,639	0.93 (0.84 to 1.02)	0.74 (0.66 to 0.82)
Subcutaneous	360	1.21 (0.44 to 1.99)	1.05 (0.26 to 1.84)
Inflammatory condition			
Rheumatoid Arthritis	15,079	0.91 (0.79 to 1.02)	0.69 (0.59 to 0.79)
Other immune mediated inflammatory diseases*	8,920	0.98 (0.83, to 1.12)	0.80 (0.67 to 0.93)

*includes psoriasis +/- arthritis, ankylosing spondylitis, reactive arthritis, lupus, systemic sclerosis, myositis, small vessel vasculitis, inflammatory bowel disease associated inflammatory arthritis.

Supplementary Table 2: Patient characteristics at the midpoint of each decile of predicted risk from a randomly selected imputation dataset.

Decile	MTX dose (mg/d)	Age (yr.)	Sex	BMI (kg/m^2)	Smoker	Alcohol	Disease	DM	CKD	IS drug	Statin	NSAID	PCM	PPI	E	BTA	Risk over 5 years
1	1.143	31	M	25.2	No	Moderate	Psoriasis or PSA	No	No	SSZ	No	No	No	No	No	No	7.1%
2	2.857	56	F	31.6	No	Ex-drinker	RA	No	No	No	No	No	No	Yes	No	No	7.4%
3	2.143	72	F	18.9	Yes	Low	RA	No	No	SSZ	No	Yes	No	No	No	No	7.5%
4	2.679	70	F	28.9	No	Low	RA	No	No	No	Yes	Yes	No	Yes	No	No	7.8%
5	2.143	71	M	26.5	No	Ex-drinker	RA	No	No	No	Yes	No	No	Yes	No	No	8.6%
6	1.429	51	F	29.0	No	Low	RA	Yes	No	No	No	No	No	No	No	No	8.8%
7	3.571	49	F	25.3	Yes	Hazardous	RA	No	No	No	No	No	No	No	No	No	9.4%
8	2.857	48	F	23.4	No	Moderate	Psoriasis or PSA	No	Yes	No	No	No	No	No	No	No	13.9%
9	1.554	59	F	36.5	No	Low	Psoriasis or PSA	No	No	No	No	Yes	No	No	No	Yes	18.9%
10	2.143	68	M	28.7	No	Low	Psoriasis or PSA	No	No	No	Yes	No	No	Yes	No	Yes	23.7%

A-E: - anti-epileptic; BMI: - Body Mass Index; BTA: - Blood Test abnormalities within 6 months of primary care methotrexate prescription; CKD: - Chronic Kidney Disease; DM: - diabetes mellitus; E: anti-epilepsy drugs; F: - female; IS – immunosuppressive drug; M: - male; MTX: - methotrexate; NSAIDS: - nonsteroidal anti-inflammatory drugs; PCM: - paracetamol; PPI: - Proton Pump Inhibitors; PSA: - psoriatic arthritis; RA: - Rheumatoid Arthritis; SSZ: - sulfasalazine;

Figure S1: Model development cohort: Study population selection

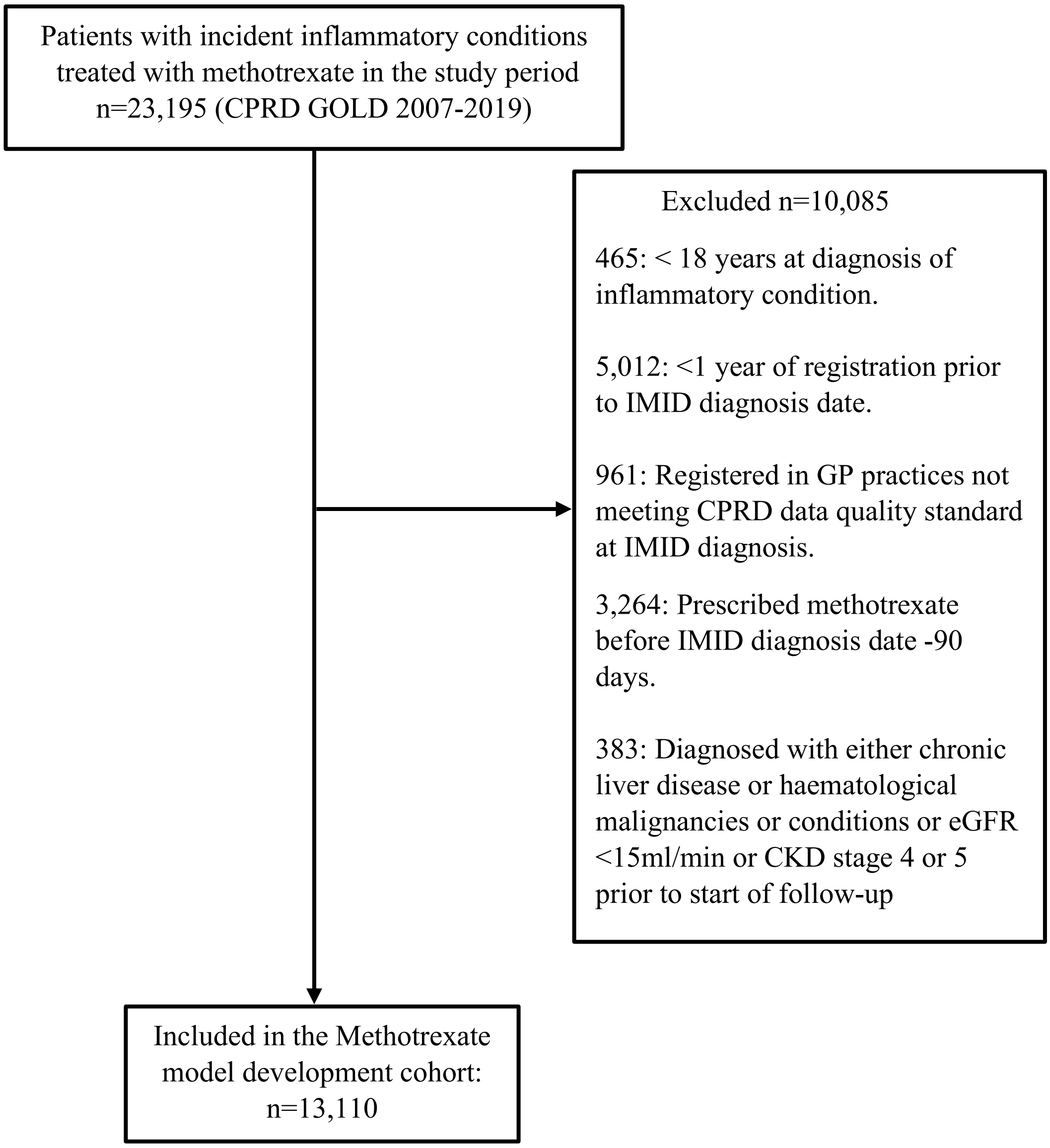

Figure S2: Model validation cohort: Study population selection

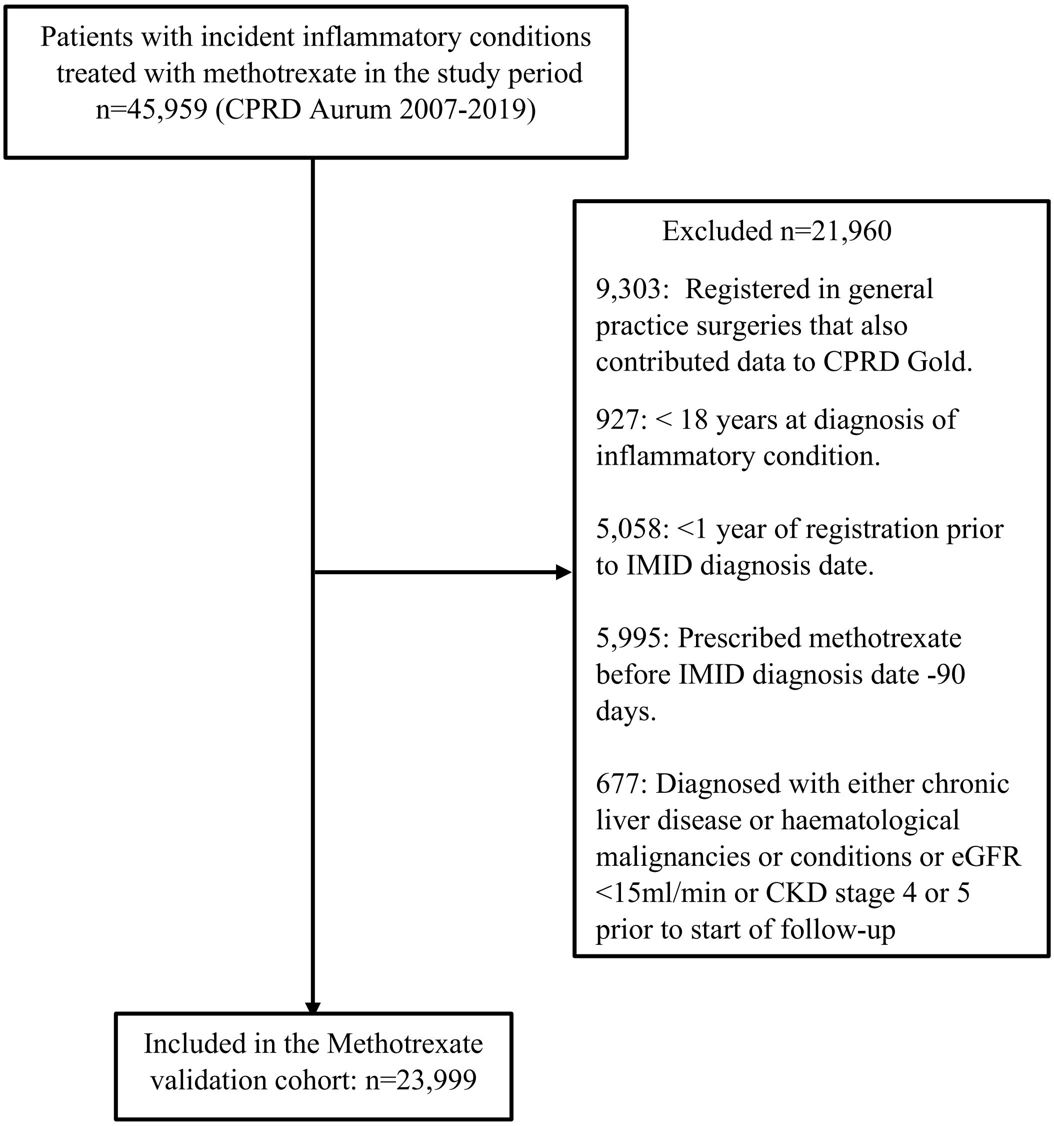

Figure S3: Calibration of a prognostic model for methotrexate discontinuation with abnormal monitoring blood-test results at 5 years in the derivation cohort

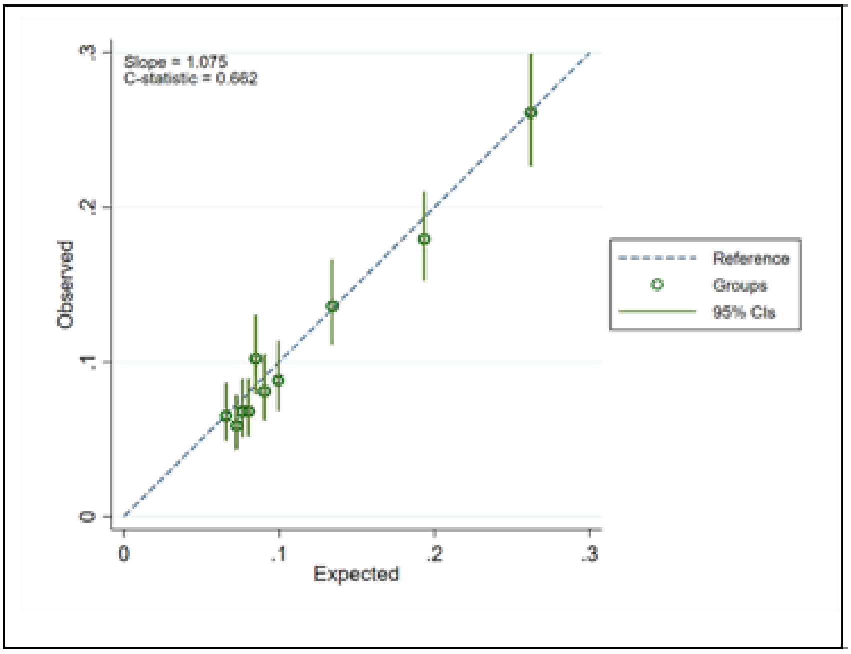

Data from a single imputed dataset; So(t=5) 0.895

Figure S4: Distribution of predicted risk of discontinuation with abnormal monitoring blood-test results in the derivation cohort at 5 years

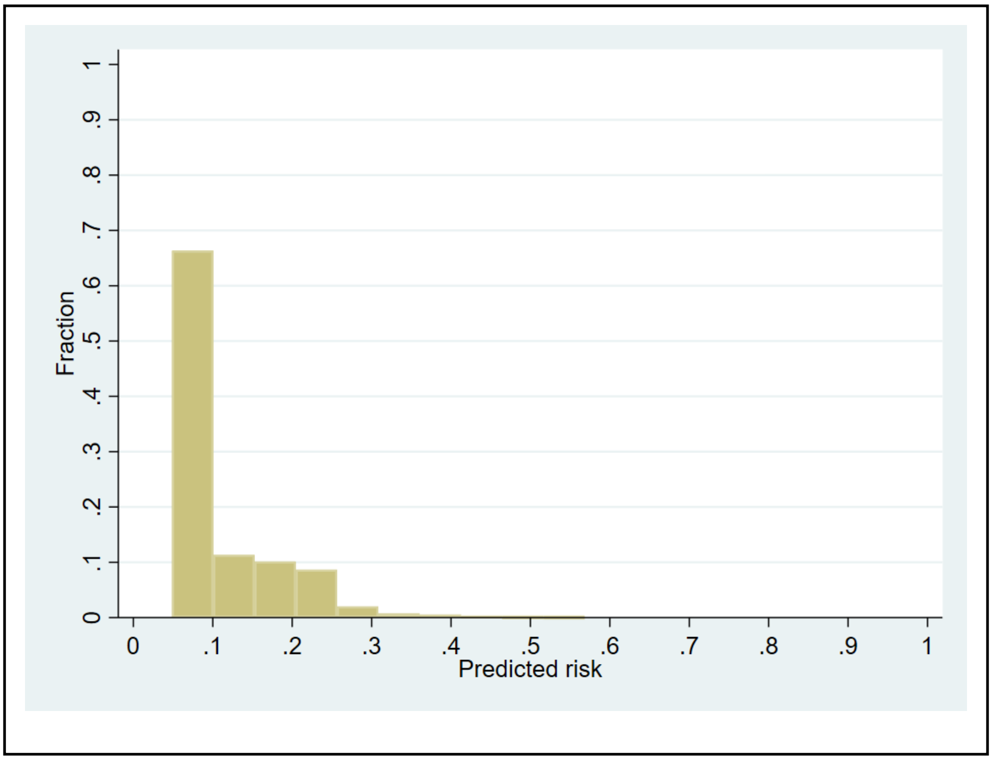

Data from a single imputed dataset

Figure S5: Calibration of a prognostic model for methotrexate discontinuation with abnormal monitoring blood-test results at 5 years in the validation cohort

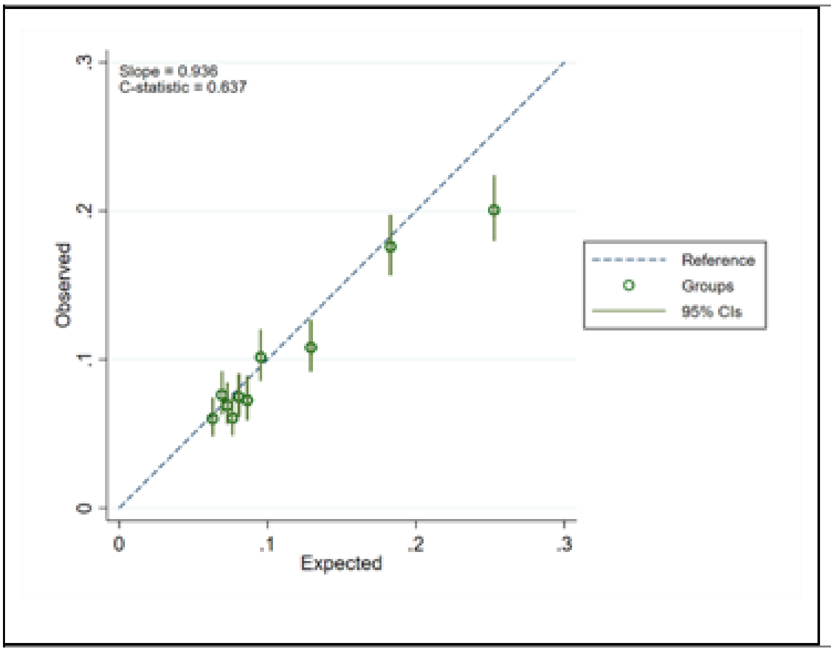

Data from a single imputed dataset; So(t=5) 0.895

Figure S6: Distribution of predicted risk of discontinuation with abnormal monitoring blood-test results in the validation cohort at 5 years

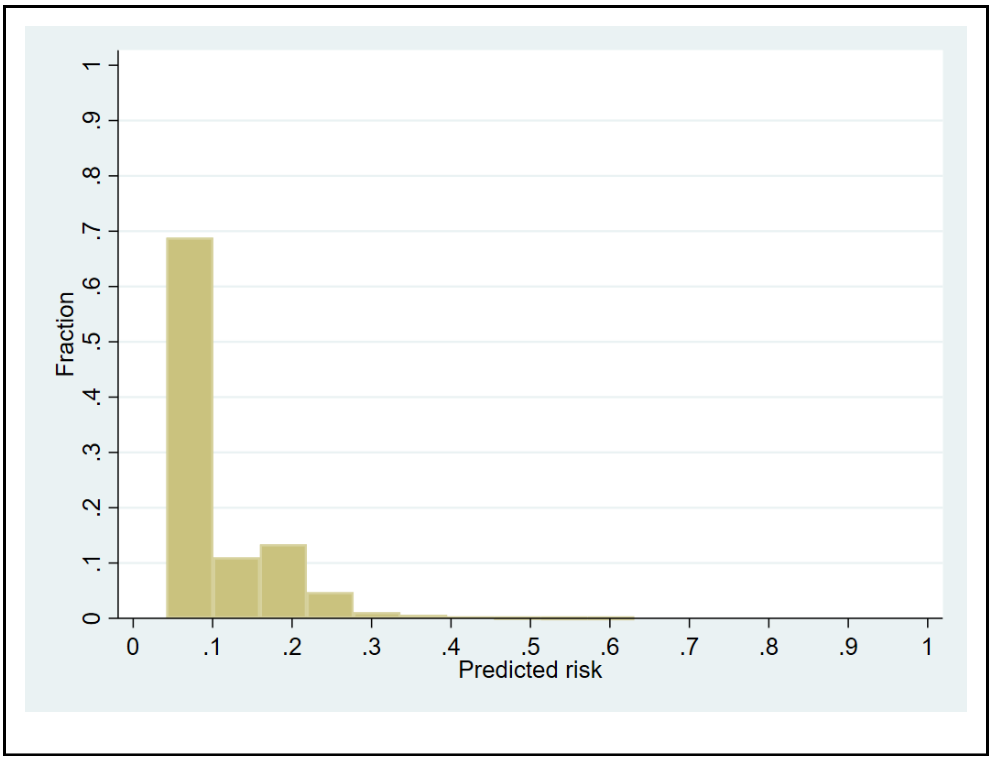

Data from a single imputed dataset

Figure S7: Calibration of a prognostic model for methotrexate discontinuation with abnormal monitoring blood-test results at 1 year in the validation cohort

a: Calibration plot

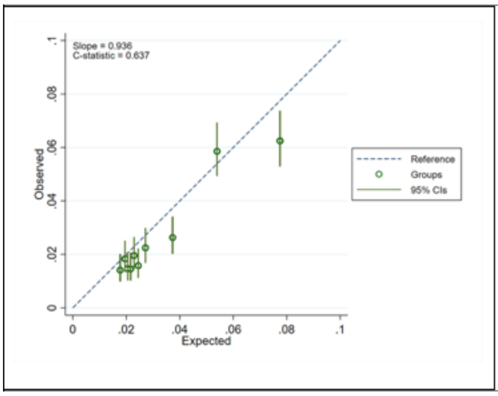

b: Smoothed calibration curve

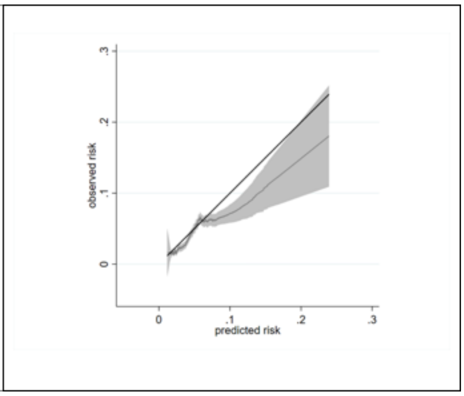

Data from a single imputed dataset; $S_o(t_{=1})$ 0.970

Figure S8: Calibration of a prognostic model for methotrexate discontinuation with abnormal monitoring blood-test results at 2 years in the validation cohort

a: Calibration plot

b: Smoothed calibration curve

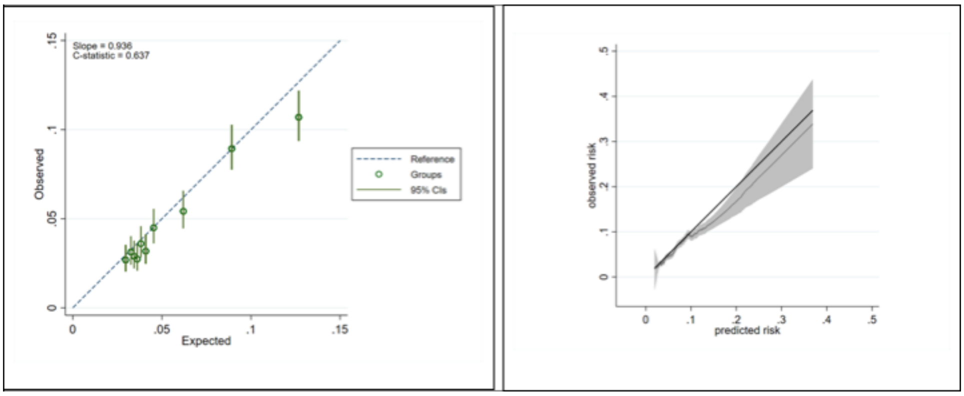

Data from a single imputed dataset; $S_o(t_{=2})$ 0.950

Figure S9: Calibration of a prognostic model for methotrexate discontinuation with abnormal monitoring blood-test results at 3 years in the validation cohort

a: Calibration plot

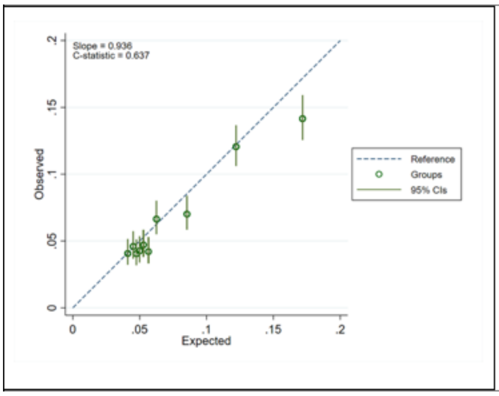

b: Smoothed calibration curve

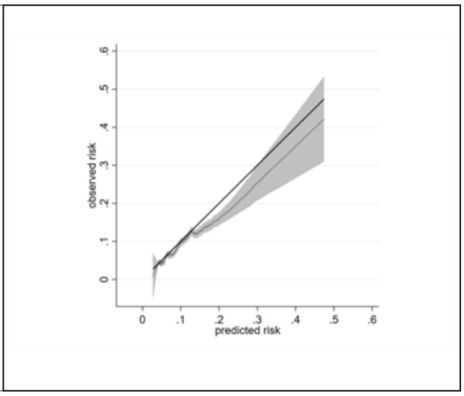

Data from a single imputed dataset; $S_o(t_{=3})$ 0.931

Figure S10: Calibration of a prognostic model for methotrexate discontinuation with abnormal monitoring blood-test results at 4 years in the validation cohort

a: Calibration plot

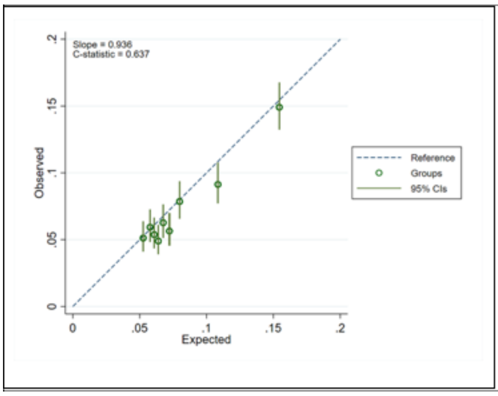

b: Smoothed calibration curve

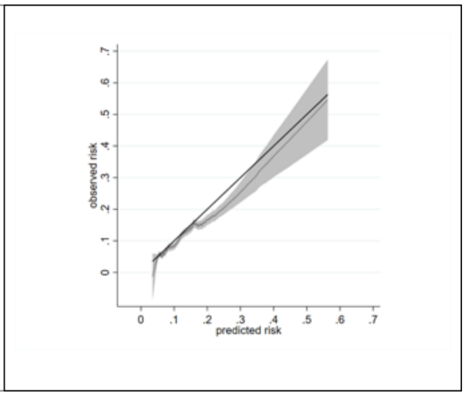

Data from a single imputed dataset; $S_o(t_{=4})$ 0.912

Figure S11: Calibration of a prognostic model for methotrexate discontinuation with abnormal monitoring blood-test results at 5 years in the validation cohort: stratified according to age

a: <60 years

b: ≥ 60 years

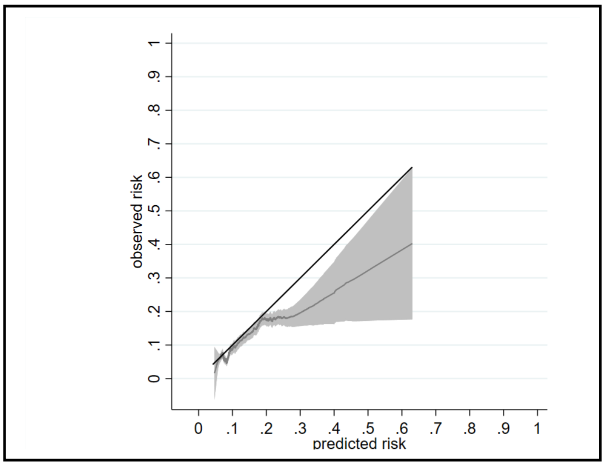

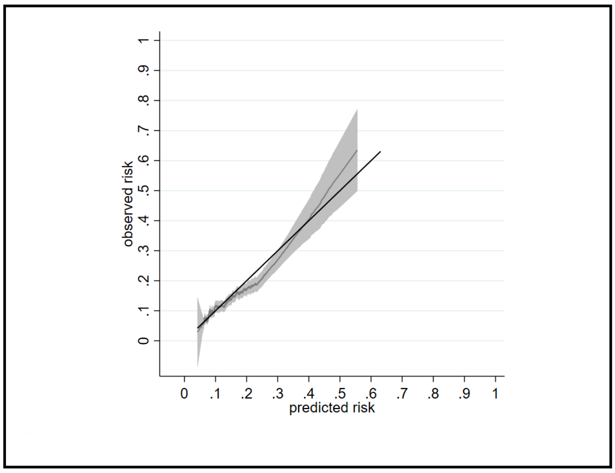

Data from a single imputed dataset; So(t=5) 0.895

Figure S12: Calibration of a prognostic model for methotrexate discontinuation with abnormal monitoring blood-test results at 5 years in the validation cohort: stratified according to inflammatory disease type

a: Rheumatoid Arthritis

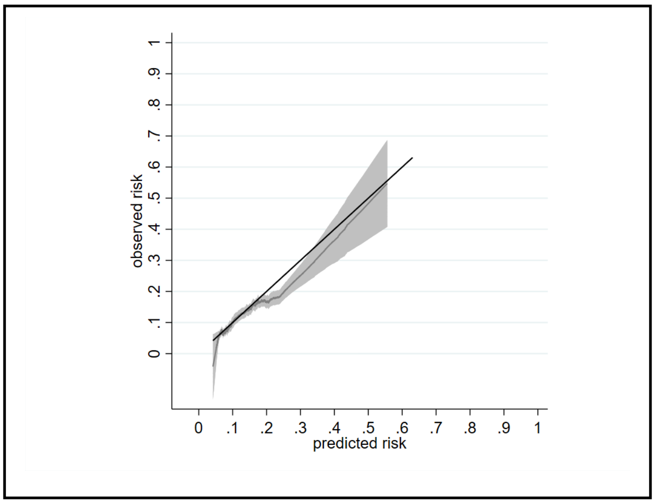

b: Other immune-mediated inflammatory diseases

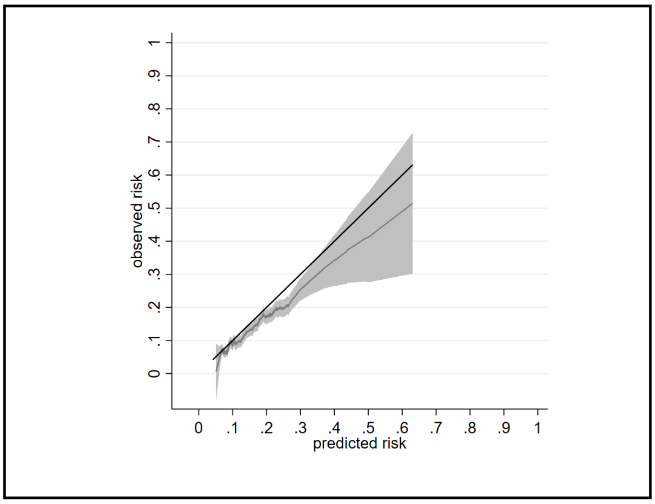

Data from a single imputed dataset; So(t=5) 0.895

Figure S13: Calibration of a prognostic model for methotrexate discontinuation with abnormal monitoring blood-test results at 5 years in the validation cohort: stratified according to route of administration.

a: Oral

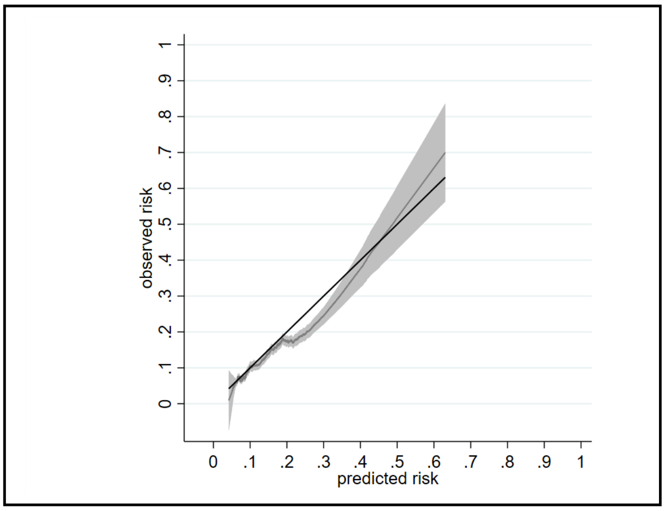

b: Subcutaneous

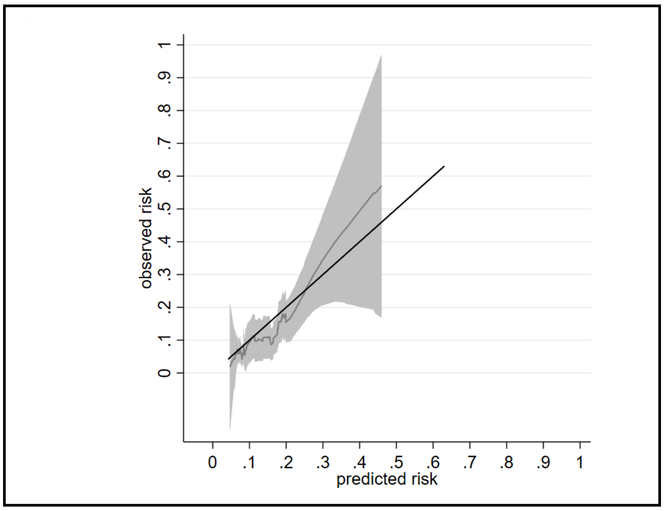

Data from a single imputed dataset; So(t=5) 0.895

Figure S14: Calibration of a prognostic model for methotrexate discontinuation with abnormal monitoring blood-test results at 5 years in the validation cohort: stratified according to methotrexate dose.

a: ≤15mg/week

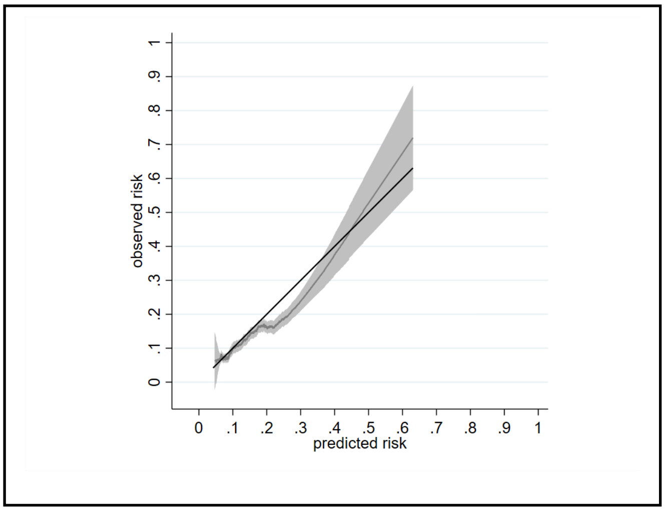

b: > 15mg/week

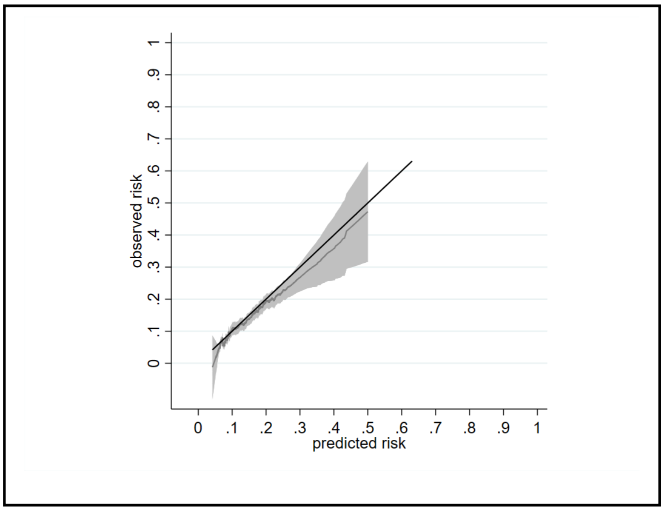

Data from a single imputed dataset; So(t=5) 0.895

Supplementary methods: list of conditions excluded from model development cohort.

medcode	readcode	readterm
1638	J615z13	Cirrhosis of liver NOS
1754	J614.00	Chronic hepatitis
1755	J614200	Chronic aggressive hepatitis
2413	A70z000	Hepatitis C
2834	A705000	Viral hepatitis C without mention of hepatic coma
2860	A703.00	Viral (serum) hepatitis B
3450	J615300	Diffuse nodular cirrhosis
4743	J612.00	Alcoholic cirrhosis of liver
5129	J623.00	Portal hypertension
5638	J616000	primary biliary cirrhosis
6015	Jyu7100	[X]Other and unspecified cirrhosis of liver
6073	C351000	Hepatolenticular degeneration (Wilson's disease)
6863	J61..00	Cirrhosis and chronic liver disease
7602	J617000	Chronic alcoholic hepatitis
7957	J614111	Autoimmune chronic active hepatitis
8206	C350012	Pigmentary cirrhosis of liver
9029	J614100	Chronic active hepatitis
9494	J616.00	Biliary cirrhosis
10539	J61z.00	Chronic liver disease NOS
10636	J624.00	Hepatorenal syndrome
11271	65V3.11	Hepatitis notification
11960	760C300	Fibreoptic endoscopic injection sclerotherapy oesoph varices
15424	J616100	Secondary biliary cirrhosis
15425	J661700	Primary sclerosing cholangitis
15489	J614z00	Chronic hepatitis NOS
16062	J62y.13	Hepatic failure
16455	J615z00	Non-alcoholic cirrhosis NOS
16725	J615.00	Cirrhosis - non alcoholic

16759	760C500	Fibreoptic endoscopic banding of oesophageal varices
17219	J635300	Toxic liver disease with chronic persistent hepatitis
17222	SP14211	Liver failure as a complication of care
17330	J613000	Alcoholic hepatic failure
18652	J63B.00	Autoimmune hepatitis
18739	J615z12	Cryptogenic cirrhosis of liver
19418	A705400	Hepatitis non A non B
19512	C310400	Glycogenosis with hepatic cirrhosis
19512	C310400	glycogenosis with hepatic cirrhosis
21769	J625.11	[X] Liver failure
22168	J601100	Subacute hepatitis - noninfective
22766	J635000	Toxic liver disease with cholestasis
22841	J615z11	Macronodular cirrhosis of liver
23511	J622.00	Hepatic coma
23578	J614000	Chronic persistent hepatitis
24813	A707000	Chronic viral hepatitis B with delta-agent
25589	C376100	Alpha-1-antitrypsin hepatitis
26319	G852200	oesophageal varices in cirrhosis of the liver
26367	A707.00	Chronic viral hepatitis
26405	J63A.00	Hepatic granulomas in sarcoidosis
26490	J601000	Subacute hepatic failure
27175	43B4.00	Hepatitis B surface antig +ve
27438	J615700	Cardiac portal cirrhosis
27565	PB62.00	Congenital cystic liver disease
27663	J63X.00	Granulomatous hepatitis, not elsewhere classified
28350	PB62100	Fibrocystic liver disease
28568	43X3.00	Hepatitis C antibody test positive
28798	J63y100	Nonspecific reactive hepatitis
28929	G857.00	Gastric varices
30586	A707200	Chronic viral hepatitis C
30729	141E.00	History of hepatitis B

30884	4J3B.00	Hepatitis C viral load
31897	J62..00	Liver abscess and sequelae of chronic liver disease
32277	A707X00	Chronic viral hepatitis, unspecified
32657	A705100	Acute delta-(super)infection of hepatitis B carrier
33316	43jG.00	Hepatitis B nucleic acid detection
33597	J61yz00	Other non-alcoholic chronic liver disease NOS
34642	Q48yz11	Congenital hepatic fibrosis
35589	4JQ3.00	Hepatitis C virus genotype
35667	J661800	Secondary sclerosing cholangitis
36107	J635100	Toxic liver disease with hepatic necrosis
36194	SP14200	Hepatic failure as a complication of care
36727	J635.00	Toxic liver disease
37521	D307000	Deficiency of coagulation factor due to liver disease
39351	J635500	Toxic liver disease with chronic active hepatitis
39945	J600011	Acute liver failure
40567	J615600	Capsular portal cirrhosis
41096	A707100	Chronic viral hepatitis B without delta-agent
41104	J635200	Toxic liver disease with acute hepatitis
41386	J635700	Acute hepatic failure due to drugs
41480	J600.00	Acute necrosis of liver
41673	J635X00	Toxic liver disease, unspecified
43404	7609300	Local ligation of oesophageal varices
44120	J635600	Toxic liver disease with fibrosis and cirrhosis of liver
44676	J615400	Fatty portal cirrhosis
44791	J661900	Sclerosing cholangitis unspecified
47257	J615.11	Portal cirrhosis
48102	J62y.00	Other sequelae of chronic liver disease
48488	J60..00	Acute and subacute liver necrosis
48879	J637.00	Hepatic veno-occlusive disease
48928	J615H00	Infectious cirrhosis NOS
53480	J614300	Recurrent hepatitis

53704	J600200	Acute yellow atrophy
53877	J614y00	Chronic hepatitis unspecified
54005	D313y00	Other specified primary thrombocytopenia
55454	J615y00	Portal cirrhosis unspecified
55561	D103100	Haemolytic anaemia due to pyruvate kinase deficiency
55637	J600z00	Acute necrosis of liver NOS
56066	4J3D.00	Hepatitis B viral load
57324	J601.00	Subacute necrosis of liver
58630	J616z00	Biliary cirrhosis NOS
60104	J61y500	Hepatic sclerosis
64451	J636.00	Central haemorrhagic necrosis of liver
64750	J635400	Toxic liver disease with chronic lobular hepatitis
65050	A704000	Viral hepatitis C with coma
65067	J60z.00	Acute and subacute liver necrosis NOS
66534	J614400	Chronic lobular hepatitis
68376	J612.11	Florid cirrhosis
69053	A702.00	Viral hepatitis B with coma
69204	J615100	Multilobular portal cirrhosis
69313	J601200	Subacute yellow atrophy
69367	J601z00	Subacute necrosis of liver NOS
72570	J638.00	Peliosis hepatis
72721	D10y.00	Other specified hereditary haemolytic anaemias
73482	J615D00	Bacterial portal cirrhosis
83476	7Q09000	Hypoplastic haemolytic and renal anaemia drugs Band 1
89717	761D800	Fibreoptic endoscopic rubber band ligitation of upper GIT varices
92909	J615500	Hypertrophic portal cirrhosis
92968	7Q09100	Hypoplastic haemolytic and renal anaemia drugs Band 2
94214	D111z00	Non-autoimmune haemolytic anaemia NOS
95926	9kZ..00	Hepatitis B screening positive - enhanced services admin
96085	9kV..00	Hepatitis C screening positive - enhanced services admin

96192	7Q05300	RSV treatment and Hepatitis C treatment drugs Band 1
97735	9kZ..11	Hepatitis B screening positive
98903	9kV..11	Hepatitis C screening positive
99308	D111y00	Other specified non-autoimmune haemolytic anaemia
99898	9kR..00	Chronic hepatitis annual review - enhanced services admin
99917	Dyu1.00	[X]Haemolytic anaemias
100253	J615C00	Xanthomatous portal cirrhosis
100388	Dyu1500	[X]Other autoimmune haemolytic anaemias
100474	J612.12	Laennec's cirrhosis
100592	J61y600	hepatic fibrosis with hepatic sclerosis
100834	7Q05200	Hepatitis B treatment drugs Band 1
102372	2126700	Hepatitis C resolved
102565	4JQD.11	Hepatitis C PCR positive
102568	4JQD.00	Hepatitis C viral ribonucleic acid PCR positive
104243	4JQF.00	Hepatitis C antigen positive
104277	14i..00	H/O hepatitis C antiviral drug therapy
104341	A70A.00	Hepatitis C genotype 1
104346	A70C.00	Hepatitis C genotype 3
104572	9NgR.00	On hepatitis C treatment plan
104579	A70G.00	Acute hepatitis C
104892	A70D.00	Hepatitis C genotype 4
105390	B937911	Myelodysplastic syndrome isolated del(5q) chromosomal abnorm
105664	A70B.00	Hepatitis C genotype 2
106025	Jyu7600	[X]Toxic liver disease, unspecified
106642	A707300	Chronic viral hepatitis B
107820	D103000	Haemolytic anaemia due to hexokinase deficiency
107896	AyuB200	[X]Chronic viral hepatitis, unspecified
108343	AyuB100	[X]Other chronic viral hepatitis
108800	J62z.00	Liver abscess and chronic liver disease causing sequelae NOS
109482	43XA.00	Hepatitis B core antibody positive

109540	J615G00	zooparasitic portal cirrhosis
110176	D103z00	Enzyme deficiency haemolytic anaemia NOS
110411	8BB5.00	12 week virologic response to hepatitis C treatment
110454	C370700	Liver disease due to cystic fibrosis
111969	9kR..11	Chronic hepatitis annual review
12479	1Z13.00	Chronic kidney disease stage 4
12585	1Z14.00	Chronic kidney disease stage 5
95122	1Z1H.00	Chronic kidney disease stage 4
95406	1Z1J.00	Chronic kidney disease stage 4
95508	1Z1K.00	Chronic kidney disease stage 5
95405	1Z1L.00	Chronic kidney disease stage 5
104963	K054.00	Chronic kidney disease stage 4
105151	K055.00	Chronic kidney disease stage 5
91911	D401.00	Functional disorders of polymorphonuclear neutrophils
38306	D401z00	Polymorphonuclear neutrophil disorder NOS
96380	D400100	Primary splenic neutropenia
42439	C391211	Thrombocytopenic eczema with immunodeficiency
877	D313.15	Thrombocytopenic purpura
21697	G756100	Thrombotic thrombocytopenic purpura
54005	D313y00	Other specified primary thrombocytopenia
5144	D313.12	Idiopathic thrombocytopenic purpura
12234	D313000	Idiopathic thrombocytopenic purpura
5181	D313012	ITP - idiopathic thrombocytopenic purpura
16420	42P2.11	Auto-immune thrombocytopenia
58906	D313111	Hereditary thrombocytopenia NEC
54005	D313y00	Other specified primary thrombocytopenia
7799	BBv..00	[M]Myelodysplastic syndrome
45143	ByuHD00	[X]Myelodysplastic syndrome, unspecified
14927	B937.14	Myelodysplasia
4561	B937W11	Myelodysplasia
104273	B677.00	Myelodysplastic and myeloproliferative disease

102712	7Q09700	Myelodysplastic syndrome drugs Band 1
105390	B937911	Myelodysplastic syndrome isolated del(5q) chromosomal abnorm
45285	B937W00	Myelodysplastic syndrome, unspecified
99917	Dyu1.00	[X]Haemolytic anaemias
100388	Dyu1500	[X]Other autoimmune haemolytic anaemias
15314	D11z.00	Acquired haemolytic anaemia NOS
50495	D112z12	Acquired haemolytic anaemia with haemoglobinuria NEC
27771	D11..00	Acquired haemolytic anaemias
39876	D110z00	Autoimmune haemolytic anaemia NOS
3818	D110.00	Autoimmune haemolytic anaemias
21369	D110.11	Coombs positive haemolysis
31734	D110400	Drug-induced autoimmune haemolytic anaemia
67088	D111400	Drug-induced haemolytic anaemia
110176	D103z00	Enzyme deficiency haemolytic anaemia NOS
71808	D102.00	Haemolytic anaemia due to glutathione metabolism disorder
107820	D103000	Haemolytic anaemia due to hexokinase deficiency
55561	D103100	Haemolytic anaemia due to pyruvate kinase deficiency
3326	D1...00	Haemolytic anaemias
18631	D1z..00	Haemolytic anaemias NOS
14698	D10z.00	Hereditary haemolytic anaemia NOS
39456	D10..00	Hereditary haemolytic anaemias
63936	D111500	Infective haemolytic anaemia
38327	D111100	Microangiopathic haemolytic anaemia
57897	D111.00	Non-autoimmune haemolytic anaemia
94214	D111z00	Non-autoimmune haemolytic anaemia NOS
39967	D1y..00	Other specified haemolytic anaemias
103151	D10yz00	Other specified hereditary haemolytic anaemia NOS
72721	D10y.00	Other specified hereditary haemolytic anaemias
99308	D111y00	Other specified non-autoimmune haemolytic anaemia
39944	D110000	Primary cold-type haemolytic anaemia

49182	D110100	Primary warm-type haemolytic anaemia
57575	D110200	Secondary cold-type haemolytic anaemia

Supplementary methods: list of conditions excluded from model validation cohort.

medcodeid	term	Original readcode	readcode	Snomedct conceptid	snomedctdescriptionid
303514011	Toxic liver disease with acute hepatitis	J6352	J635200	197358007	303514011
325079010	Liver failure as a complication of care	SP142-1	SP14211	213230009	325079010
303614019	Primary sclerosing cholangitis	J6617	J661700	197441003	303614019
2159899016	Autoimmune hepatitis	J63B	J63B.00	408335007	2159899016
2534188017	Acute hepatic failure due to drugs	J6357	J635700	413438002	2534188017
303525019	Hepatic granulomas in sarcoidosis	J63A	J63A.00	197368002	303525019
303466016	Liver abscess and sequelae of chronic liver disease	J62	J62..00	197324004	303466016
303517016	Toxic liver disease with chronic active hepatitis	J6355	J635500	197361008	303517016
411809016	Hepatitis notification	65V3-1	65V3.11	275847004	411809016
303440019	Zooparasitic portal cirrhosis	J615G	J615G00	197306001	303440019
303410016	Chronic hepatitis NOS	J614z	J614z00	76783007	127487019
55851000006118	Xanthomatous portal cirrhosis	J615C	J615C00	271440004	406232019
96432016	Peliosis hepatis	J638	J638.00	58008004	96432016
451442010	Oesophageal varices in cirrhosis of the liver	G8522	G852200	308129003	451442010
325080013	Hepatic failure as a complication of care	SP142	SP14200	213230009	325080013
354081000000114	Hepatitis B viral load	4J3D	4J3D.00	222531000000101	354081000000114
353625019	Hepatic sclerosis	J61y5	J61y500	235899008	353625019
303516013	Toxic liver disease with chronic lobular hepatitis	J6354	J635400	197360009	303516013
60332100000 6118	Cryptogenic cirrhosis of liver	J615z-2	J615z12	89580002	148522018
95368015	Chronic lobular hepatitis	J6144	J614400	57339008	95368015

303518014	Toxic liver disease with fibrosis and cirrhosis of liver	J6356	J635600	197362001	303518014
303511015	Toxic liver disease with cholestasis	J6350	J635000	197355005	303511015
820481000006115	Hepatic veno-occlusive disease	J637	J637.00	65617004	3028711017
1232261014	Chronic viral hepatitis B	A7073	A707300	61977001	1232261014
683211000000113	Chronic hepatitis annual review	9kR-1	9kR..11	362421000000102	683211000000113
1739601000000111	Hepatitis C PCR positive	4JQD-1	4JQD.11	760421000000100	1739601000000111
303409014	Chronic hepatitis unspecified	J614y	J614y00	76783007	127487019
303521011	Central haemorrhagic necrosis of liver	J636	J636.00	197364000	303521011
806841000006117	Granulomatous hepatitis, not elsewhere classified	J63X	J63X.00	86514004	143474019
669601000000113	Hepatitis B screening positive	9kZ-1	9kZ..11	356121000000100	669601000000113
286879013	Chronic viral hepatitis B without delta-agent	A7071	A707100	186639003	286879013
122921000006118	Subacute hepatitis - noninfective	J6011	J601100	72445008	1233507016
303512010	Toxic liver disease with hepatic necrosis	J6351	J635100	197356006	303512010
2156971000000110	H/O hepatitis C antiviral drug therapy	14i	14i..00	829691000000100	2156971000000110
353575013	Acute delta-(super)infection of hepatitis B carrier	A7051	A705100	235865005	353575013
1786709019	Pigmentary cirrhosis of liver	C3500-2	C350012	399126000	1786709019
731231000006114	Macronodular cirrhosis of liver	J615z-1	J615z11	43904005	73201018
821141000006113	Hepatolenticular degeneration (Wilson's disease)	C3510	C351000	88518009	146760018
303781011	[X]Other and unspecified cirrhosis of liver	Jyu71	Jyu7100	19943007	33568015
286862019	Viral hepatitis B with coma	A702	A702.00	26206000	3008554017

396394015	Other sequelae of chronic liver disease	J62y	J62y.00	235856003	353559010
61061000006116	Viral (serum) hepatitis B	A703	A703.00	66071002	109733012
303442010	Portal cirrhosis unspecified	J615y	J615y00	419728003	2576980018
353613017	Cirrhosis of liver NOS	J615z-3	J615z13	19943007	33568015
69895016	Chronic persistent hepatitis	J6140	J614000	41889008	69895016
303464018	Other non-alcoholic chronic liver disease NOS	J61yz	J61yz00	328383001	467154010
353627010	Hepatic fibrosis with hepatic sclerosis	J61y6	J61y600	235901004	353627010
303518014	Toxic liver disease with fibrosis and cirrhosis of liver	J6356	J635600	197362001	303518014
603321000006118	Cryptogenic cirrhosis of liver	J615z-2	J615z12	89580002	148522018
451124014	Chronic alcoholic hepatitis	J6170	J617000	307757001	451124014
4044018	Biliary cirrhosis	J616	J616.00	1761006	4044018
303393017	Cirrhosis and chronic liver disease	J61	J61..00	197279005	303393017
353613017	Cirrhosis of liver NOS	J615z-3	J615z13	19943007	33568015
293605014	Alpha-1-antitrypsin hepatitis	C3761	C376100	190944000	293605014
303441015	Infectious cirrhosis NOS	J615H	J615H00	235896001	353622016
303517016	Toxic liver disease with chronic active hepatitis	J6355	J635500	197361008	303517016
303437019	Bacterial portal cirrhosis	J615D	J615D00	197303009	303437019
2576980018	Portal cirrhosis	J615-1	J615.11	419728003	2576980018
120980011	Hepatic coma	J622	J622.00	72836002	120980011
820791000006114	Hepatitis B surface antig +ve	43B4	43B4.00	165806002	257840015
286871011	Viral hepatitis C without mention of hepatic coma	A7050	A705000	50711007	84513012
353595019	Alcoholic hepatic failure	J6130	J613000	235881000	353595019
303510019	Toxic liver disease	J635	J635.00	197354009	303510019
2146521000000117	Hepatitis C genotype 4	A70D	A70D.00	824881000000102	2146521000000117

350841000000119	Hepatitis B treatment drugs Band 1	7Q052	7Q05200	221021000000103	350841000000119
2675247013	Liver disease due to cystic fibrosis	C3707	C370700	427022004	2675247013
303407011	Recurrent hepatitis	J6143	J614300	197286002	303407011
2693857014	History of hepatitis B	141E	141E.00	429721005	2693857014
303515012	Toxic liver disease with chronic persistent hepatitis	J6353	J635300	197359004	303515012
1680731000006114	Hepatitis B screening positive - enhanced services admin	9kZ	9kZ..00	356121000000100	728581000000110
2155841000000118	12 week virologic response to hepatitis C treatment	8BB5	8BB5.00	829241000000108	2155841000000118
557081000006110	Chronic viral hepatitis, unspecified	A707X	A707X00	10295004	17930015
303781011	[X]Other and unspecified cirrhosis of liver	Jyu71	Jyu7100	19943007	33568015
303515012	Toxic liver disease with chronic persistent hepatitis	J6353	J635300	197359004	303515012
353580016	Chronic viral hepatitis B with delta-agent	A7070	A707000	235869004	353580016
288170016	[X]Chronic viral hepatitis, unspecified	AyuB2	AyuB200	10295004	17930015
803961000006119	Glycogenosis with hepatic cirrhosis	C3104	C310400	29633007	49567015
459095018	Hepatitis C antibody test positive	43X3	43X3.00	314706002	459095018
396390012	Multilobular portal cirrhosis	J6151	J615100	266469006	396390012
2146441000000113	Hepatitis C genotype 1	A70A	A70A.00	824841000000105	2146441000000113
303516013	Toxic liver disease with chronic lobular hepatitis	J6354	J635400	197360009	303516013
821081000006115	Hepatitis non A non B	A7054	A705400	186634008	286874015
553311000006111	Chronic aggressive hepatitis	J6142	J614200	197284004	303401012
1680661000006114	Hepatitis C screening positive - enhanced services admin	9kV	9kV..00	362751000000101	683911000000119
288169017	[X]Other chronic viral hepatitis	AyuB1	AyuB100	10295004	17930015

476611000006116	Alcoholic cirrhosis of liver	J612	J612.00	420054005	2920441014
17930015	Chronic viral hepatitis	A707	A707.00	10295004	17930015
557071000006112	Chronic viral hepatitis C	A7072	A707200	128302006	206586011
493707012	Hypertrophic portal cirrhosis	J6155	J615500	43904005	493707012
1560921000006115	RSV treatment and Hepatitis C treatment drugs Band 1	7Q053	7Q05300	221061000000106	1057891000000114
303393017	Cirrhosis and chronic liver disease	J61	J61..00	197279005	303393017
353567019	Nonspecific reactive hepatitis	J63y1	J63y100	235858002	353567019
1766481000006112	Hepatitis C viral ribonucleic acid PCR positive	4JQD	4JQD.00	760421000000100	1681741000000116
99171000006113	Toxic liver disease, unspecified	J635X	J635X00	197354009	303510019
303401012	Chronic active hepatitis	J6141	J614100	197284004	303401012
126721013	Florid cirrhosis	J612-1	J612.11	76301009	126721013
2118321000000118	Hepatitis C antigen positive	4JQF	4JQF.00	812181000000106	2118321000000118
303489012	Liver abscess and chronic liver disease causing sequelae NOS	J62z	J62z.00	197324004	303466016
1680591000006117	Chronic hepatitis annual review - enhanced services admin	9kR	9kR..00	362421000000102	683201000000111
303419015	Diffuse nodular cirrhosis	J6153	J615300	197293003	303419015
1136251000000110	Hepatitis B core antibody positive	43XA	43XA.00	736687002	3521926012
803961000006119	Glycogenosis with hepatic cirrhosis	C3104	C310400	29633007	49567015
1783609017	Hepatitis C virus genotype	4JQ3	4JQ3.00	992771000000100	2581311000000111
1488891017	Hepatitis C viral load	4J3B	4J3B.00	992751000000109	2576171000000118
303387013	Subacute necrosis of liver	J601	J601.00	197274000	303387013
303617014	Sclerosing cholangitis unspecified	J6619	J661900	235917005	353654013
2157051000000119	On hepatitis C treatment plan	9NgR	9NgR.00	829731000000106	2157051000000119

221111000000114	Cirrhosis - non alcoholic	J615	J615.00	266468003	396389015
84516016	Hepatitis C	A70z0	A70z000	50711007	84516016
303514011	Toxic liver disease with acute hepatitis	J6352	J635200	197358007	303514011
1686831000000111	Hepatitis C resolved	21267	2126700	761381000000102	1686831000000111
1786360015	Hepatitis B nucleic acid detection	43jG	43jG.00	1031571000000101	2595441000000119
303512010	Toxic liver disease with hepatic necrosis	J6351	J635100	197356006	303512010
303404016	Autoimmune chronic active hepatitis	J6141-1	J614111	197284004	303404016
2579818016	Laennec's cirrhosis	J612-2	J612.12	419728003	2576980018
303391015	Subacute necrosis of liver NOS	J601z	J601z00	235856003	353559010
683911000000119	Hepatitis C screening positive	9kV-1	9kV..11	362751000000101	683911000000119
303786018	[X]Toxic liver disease, unspecified	Jyu76	Jyu7600	197354009	303510019
359011000006113	[X] Liver failure	J625-1	J625.11	59927004	498583014
303616017	Secondary sclerosing cholangitis	J6618	J661800	197442005	303616017
2146501000000114	Hepatitis C genotype 3	A70C	A70C.00	824871000000104	2146501000000114
286868015	Viral hepatitis C with coma	A7040	A704000	186628001	286868015
476611000006116	Alcoholic cirrhosis of liver	J612	J612.00	420054005	2920441014
99549014	Hepatic failure	J62y-3	J62y.13	59927004	99549014
85441018	Hepatorenal syndrome	J624	J624.00	51292008	85441018
303388015	Subacute hepatic failure	J6010	J601000	197275004	303388015
396391011	Cardiac portal cirrhosis	J6157	J615700	266470007	396391011
303453016	Biliary cirrhosis NOS	J616z	J616z00	1761006	4044018
52980015	Primary biliary cirrhosis	J6160	J616000	31712002	52980015
2146461000000114	Hepatitis C genotype 2	A70B	A70B.00	824851000000108	2146461000000114

127487019	Chronic hepatitis	J614	J614.00	76783007	127487019
353576014	Acute hepatitis C	A70G	A70G.00	235866006	353576014
303420014	Fatty portal cirrhosis	J6154	J615400	197294009	303420014
303465017	Chronic liver disease NOS	J61z	J61z.00	328383001	467154010
396393014	Non-alcoholic cirrhosis NOS	J615z	J615z00	266468003	396389015
132070012	Congenital hepatic fibrosis	Q48yz-1	Q48yz11	79607001	132070012
21289010	Secondary biliary cirrhosis	J6161	J616100	12368000	21289010
303425016	Capsular portal cirrhosis	J6156	J615600	197296006	303425016
906081000006114	[RFC] Cirrhosis	HNG0090		906081000006105	906081000006114
906001000006117	[RFC] Liver failure	HNG0084		906001000006101	906001000006117
1761241000006114	[SHHAPT] Viral hepatitis B (HbsAg positive) - first diagnosis	C13-SHHAPT		1761241000006105	1761241000006114
1761251000006111	[SHHAPT] Viral hepatitis C - first diagnosis	C14-SHHAPT		1761251000006107	1761251000006111
459860017	[V]Hepatitis B carrier	ZV02B	ZV02B00	235871004	353582012
2474329018	[V]Hepatitis C carrier	ZV02C	ZV02C00	235872006	353583019
460192013	[V]Viral hepatitis carrier	ZV026	ZV02600	170489004	264367013
303787010	[X]Granulomatous hepatitis, not elsewhere classified	Jyu77	Jyu7700	86514004	143474019
2474731013	[X]Hepatopulmonary syndrome	Hyu52	Hyu5200	371067004	1209775018
6947461000006115	Alcoholic cirrhosis	^ESCTAL694746		420054005	2576981019
353594015	Alcoholic fibrosis and sclerosis of liver	J6120	J612000	235880004	353594015
6947471000006110	Alcoholic liver cirrhosis	^ESCTAL694747		420054005	2912539019
6947471000006110	Alcoholic liver cirrhosis	^ESCTAL694747		420054005	2912539019
3165681000006118	Alcoholic liver disease	^ESCTAL316568		41309000	68909012
3165691000006115	ALD - Alcoholic liver disease	^ESCTAL316569		41309000	492959019

4875710000 06116	Antigen Positive Viral Hepatitis B	C13-KC60		487571000 006100	487571000006 116
6617771000 006112	Bronze cirrhosis	^ESCTBR 661777		399126000	1778586012
6617781000 006110	Bronzed cirrhosis	^ESCTBR 661778		399126000	1786708010
4787131000 006111	CAH - Chronic active hepatitis	^ESCTCA 478713		197284004	303403010
4787151000 006116	CAH - Chronic aggressive hepatitis	^ESCTCA 478715		197284004	303405015
3713561000 006119	Cardiac cirrhosis	^ESCTCA 371356		74669004	123997016
303438012	Cardituberculous cirrhosis	J615E	J615E00	301811001	443293016
4787621000 006118	Central hemorrhagic necrosis of liver	^ESCTCE 478762		197364000	303520012
3937821000 006118	Cerebrohepatorenal syndrome	^ESCTCE 393782		88469006	146683010
3748781000 006119	CH - Chronic hepatitis	^ESCTCH 374878		76783007	503370016
2526441000 006114	Cholangitic cirrhosis	^ESCTCH 252644		1761006	4045017
2526451000 006111	Cholestatic cirrhosis	^ESCTCH 252645		1761006	4046016
3217881000 006113	Chronic idiopathic jaundice with pigmented liver	^ESCTCH 321788		44553005	74312010
3059381000 006110	Chronic passive congestion of liver	^ESCTCH 305938		34736002	57977012
5558810000 06114	Chronic passive liver congestion	J630	J630.00	34736002	57977012
3507191000 006112	Chronic type B viral hepatitis	^ESCTCH 350719		61977001	103019010
4407011000 006114	Chronic type C viral hepatitis	^ESCTCH 440701		128302006	206585010
5086651000 006118	Chronic viral hepatitis B with hepatitis D	^ESCTCH 508665		235869004	353579019
3828201000 006118	Chronic yellow atrophy of liver	^ESCTCH 382820		81675001	135482018
504762019	Chronic yellow liver atrophy	J61y0	J61y000	81675001	504762019

8242561000006112	Cirrhosis associated with cystic fibrosis	^ESCTCI824256		776981000000103	1735771000000114
1763641000006113	Cirrhosis of liver due to cystic fibrosis	EMISNQCI2		1763641000006109	1763641000006113
1763641000006113	Cirrhosis of liver due to cystic fibrosis	EMISNQCI2		1763641000006109	1763641000006113
5493051000006111	Cirrhosis of liver not due to alcohol	^ESCTCI549305		266468003	3038846010
5493051000006111	Cirrhosis of liver not due to alcohol	^ESCTCI549305		266468003	3038846010
5524681000006114	Cirrhosis secondary to cholestasis	^ESCTCI552468		271440004	406232019
2598561000006116	Cirrhosis-familial with pulmonary hypertension	^ESCTCI259856		6183001	499131018
5493041000006114	Cirrhosis, nonalcoholic	^ESCTCI549304		266468003	2921132017
2816681000006110	CL - Cirrhosis of liver	^ESCTCL281668		19943007	480137013
2816681000006110	CL - Cirrhosis of liver	^ESCTCL281668		19943007	480137013
3431801000006116	CLH - Chronic lobular hepatitis	^ESCTCL343180		57339008	1231718013
538081000000115	Compensation for liver failure	7L1f	7L1f.00	773411000000107	1726971000000115
538101000000114	Compensation for liver failure NOS	7L1fz	7L1fz00	773411000000107	1726971000000115
3713571000006114	Congestive cirrhosis	^ESCTCO371357		74669004	123999018
123999018	Congestive cirrhosis	J6157-1	J615711	74669004	123997016
3175071000006117	CPH - Chronic persistent hepatitis	^ESCTCP317507		41889008	1229804015
5992721000006114	Esophageal varices in alcoholic cirrhosis of the liver	^ESCTES599272		309783001	453266011
5992721000006114	Esophageal varices in alcoholic cirrhosis of the liver	^ESCTES599272		309783001	453266011
5975531000006117	Esophageal varices in cirrhosis of the liver	^ESCTES597553		308129003	451443017

5975531000006117	Esophageal varices in cirrhosis of the liver	^ESCTES597553		308129003	451443017
8029551000006113	H/O hepatitis C	^ESCTHO802955		93871000119101	2722791000000116
994011000006111	H/O: hepatitis B	EMISNQHO3		994011000006107	994011000006111
3010831000006117	Hanot's cirrhosis	^ESCTHA301083		31712002	196875018
2720851000006112	HE - Hepatic encephalopathy	^ESCTHE272085		13920009	475600012
2816671000006112	Hepatic cirrhosis	^ESCTHE281667		19943007	33572016
2816671000006112	Hepatic cirrhosis	^ESCTHE281667		19943007	33572016
71921000033118	Hepatitis B	DRGC812		71921000033102	71921000033118
3574421000006117	Hepatitis B	^ESCTHE357442		66071002	109735017
2015131000006111	Hepatitis B deoxyribonucleic acid detection assay positive	EMISNQHE217		2015131000006107	2015131000006111
3574441000006112	Hepatitis B infection	^ESCTHE357444		66071002	2536443012
1977011000006113	Hepatitis B surface antibody test positive	EMISNQHE178		1977011000006109	1977011000006113
12758661000006113	Hepatitis B with delta agent coinfection	^ESCT1275866		424460009	2647868011
12758651000006111	Hepatitis B with delta agent superinfection	^ESCT1275865		424460009	2647867018
12722521000006116	Hepatitis B with hepatitis D superinfection	^ESCT1272252		424460009	2643530016
4596181000006111	Hepatitis Be antigen present	^ESCTHE459618		165807006	2820207017
939481000006116	Hepatitis C carrier	EMISNQHE6		939481000006100	939481000006116
2146541000000112	Hepatitis C genotype 5	A70E	A70E.00	824891000000100	2146541000000112
2146561000000113	Hepatitis C genotype 6	A70F	A70F.00	824901000000104	2146561000000113

1676391000006116	Hepatitis C PCR positive	EMISNQHE29		1676391000006100	1676391000006116
9940210000006115	Hepatitis C positive	EMISNQHE11		994021000006104	994021000006115
2004311000006118	Hepatitis C post-test counselling	EMISNQHE190		2004311000006102	2004311000006118
416052019	Hepatitis C status	2J1	2J1..00	278973007	416052019
12758671000006118	Hepatitis D infection	^ESCT1275867		424460009	2647869015
4742611000006119	Hepatitis non-A non-B	^ESCTHE474261		186634008	286874015
3938581000006118	Hepatocerebral degeneration	^ESCTHE393858		88518009	508067014
2720841000006110	Hepatocerebral encephalopathy	^ESCTHE272084		13920009	23736018
3684461000006114	Hepatocerebral intoxication	^ESCTHE368446		72836002	120981010
3938561000006111	Hepatolenticular degeneration syndrome	^ESCTHE393856		88518009	146761019
3331291000006117	Hepatorenal failure	^ESCTHE333129		51292008	495986010
325081012	Hepatorenal syndrome as a complication of care	SP143	SP14300	213231008	325081012
3331281000006115	HRF - Hepatorenal failure	^ESCTHR333128		51292008	495985014
2598551000006118	ICC - Indian childhood cirrhosis	^ESCTIC259855		6183001	499130017
11267010	Indian childhood cirrhosis	J6158-2	J615812	6183001	11267010
396392016	Juvenile portal cirrhosis	J6158	J615800	266471006	396392016
5086991000006115	Laennec cirrhosis, non-alcoholic	^ESCTLA508699		235895002	2837783011
6941931000006112	Laennec's cirrhosis	^ESCTLA694193		419728003	2579818016
353621011	Laennec's cirrhosis, non-alcoholic	J615z-4	J615z14	235895002	353621011
353596018	Liver failure NOS	J62y-2	J62y.12	59927004	498583014
3206871000006111	Macronodular cirrhosis	^ESCTMA320687		43904005	73201018

4786981000 006112	Massive hepatic necrosis	^ESCTMA 478698		197269008	303378016
2753761000 006112	Mixed micro AND macronodular cirrhosis	^ESCTMI2 75376		15999000	27083014
477604016	Mixed portal cirrhosis	J6152	J615200	15999000	477604016
3206901000 006111	Multilobar cirrhosis	^ESCTMU 320690		43904005	2647101016
1587001000 006114	OS other transjugular intrahepatic ops on blood vessel liver	780Gy	780Gy00	233751000 000108	376981000000 115
1587001000 006114	OS other transjugular intrahepatic ops on blood vessel liver	780Gy	780Gy00	233751000 000108	376981000000 115
1587041000 006111	OS other transluminal operations on blood vessel of liver	780Hy	780Hy00	265439007	394238015
1545731000 006115	OS transjugular intrahepatic operations blood vessel liver	780Dy	780Dy00	233751000 000108	376981000000 115
1545731000 006115	OS transjugular intrahepatic operations blood vessel liver	780Dy	780Dy00	233751000 000108	376981000000 115
1545791000 006116	OS transluminal insertion of prosthesis blood vessel liver	780Ey	780Ey00	429066008	2692482012
1586971000 006119	Oth transjugular intrahepatic operations blood vessel liver	780G	780G.00	233751000 000108	376981000000 115
1586971000 006119	Oth transjugular intrahepatic operations blood vessel liver	780G	780G.00	233751000 000108	376981000000 115
1587011000 006112	Other transjugular intrahepatic op on blood vessel liver NOS	780Gz	780Gz00	233751000 000108	376981000000 115
6941941000 006119	PC - Portal cirrhosis	^ESCTPC 694194		419728003	2579819012
303433015	Pigmentary portal cirrhosis	J6159	J615900	197299004	303433015
2261610000 06113	Pipe-stem portal cirrhosis	J615A	J615A00	197300007	303434014
4787221000 006115	Pipestem portal cirrhosis	^ESCTPI4 78722		197300007	303434014
3206891000 006112	PNC - Postnecrotic cirrhosis	^ESCTPN 320689		43904005	493708019
303458013	Portal fibrosis without cirrhosis	J61y3	J61y300	197316009	303458013
2118210000 06111	Postnecrotic cirrhosis of liver	J6151-1	J615111	43904005	73201018

2824431000006111	Progressive neuronal degeneration with liver cirrhosis	^ESCTPR282443		20415001	1222436018
4787861000006113	PSC - Primary sclerosing cholangitis	^ESCTPS478786		197441003	303615018
2765801000006114	PTC - Percutaneous transhepatic cholangiogram	^ESCTPT276580		16747000	477927014
4787481000006117	Quaternary syphilitic hepatitis	^ESCTQU478748		197347003	2646441011
270232016	Replacement of previous liver transplant	78002	7800200	174427006	270232016
3813641000006119	Secondary syphilis of liver	^ESCTSE381364		80770009	133977016
133978014	Secondary syphilitic hepatitis	A9161	A916100	80770009	133978014
4787061000006118	Subacute hepatic necrosis	^ESCTSU478706		197274000	303386016
3678081000006113	Subacute non-infective hepatitis	^ESCTSU367808		72445008	1233507016
3678071000006110	Subacute noninfective hepatitis	^ESCTSU367807		72445008	120350013
4787101000006115	Subacute yellow atrophy of liver	^ESCTSU478710		197276003	303390019
3659301000006110	Subtotal hepatectomy	^ESCTSU365930		71273005	118368011
142629014	Syphilis of liver	A953	A953.00	86028001	142629014
303439016	Syphilitic portal cirrhosis	J615F	J615F00	197305002	303439016
5050891000006117	TIPS - Transjugular intrahepatic portosystemic shunt	^ESCTTI505089		233445008	349796018
864291000006118	Total hepatectomy	7800-99	7800.99	18027006	864291000006118
303435010	Toxic portal cirrhosis	J615B	J615B00	197301006	303435010
1545691000006111	Transjugular intrahepatic creation of portosystemic shunt	780D3	780D300	233445008	349797010
1586991000006118	Transjugular intrahepatic ins stent graft into portal vein	780G1	780G100	429080001	2694300016
1586981000006116	Transjugular intrahepatic insertion stent into portal vein	780G0	780G000	280921000000100	483051000000116

1545741000006113	Transjugular intrahepatic operations blood vessel liver NOS	780Dz	780Dz00	233751000000108	376981000000115
1545651000006117	Transjugular intrahepatic operations blood vessel of liver	780D	780D.00	233751000000108	376981000000115
3574401000006110	Type B viral hepatitis	^ESCTTY357440		66071002	109732019
3321231000006119	Type C viral hepatitis	^ESCTTY332123		50711007	84515017
303414013	Unilobular portal cirrhosis	J6150	J615000	197291001	303414013
3566811000006113	Veno-occlusive disease of the liver	^ESCTVE356681		65617004	109013019
12758681000006115	Viral hepatitis B with delta agent superinfection	^ESCT1275868		424460009	2647870019
2919481000006111	Viral hepatitis B with hepatic coma	^ESCTVI291948		26206000	43901018
12758641000006114	Viral hepatitis B with hepatitis D superinfection	^ESCT1275864		424460009	2647866010
925431000006118	Viral hepatitis carrier	PCNQVI1		925431000006102	925431000006118
264367013	Viral hepatitis carrier	65Q7	65Q7.00	170489004	264367013
1805791000006114	Viral hepatitis D	EMISNQVI15		424460009	1805791000006114
12758691000006117	Viral hepatitis delta	^ESCT1275869		424460009	619151000000117
3574451000006114	Viral hepatitis type B	^ESCTVI357445		66071002	2983616016
3321251000006114	Viral hepatitis type C	^ESCTVI332125		50711007	2983590017
4787261000006114	Zooparasitic portal cirrhosis	^ESCTZO478726		197306001	303440019
		J625	J625.00	59927004	99549014
294079010	Other specified hereditary haemolytic anaemias	D10y	D10y.00	38911009	65264014
294115013	Non-autoimmune haemolytic anaemia NOS	D111z	D111z00	191216004	294105019
294093015	Primary cold-type haemolytic anaemia	D1100	D110000	191210005	294093015

123869011	Secondary thrombocytopenia	D314	D314.00	154826009	240835016
399239014	Neutropenia due to irradiation	D4003-2	D400312	267540007	399239014
294082017	Other specified hereditary haemolytic anaemia NOS	D10yz	D10yz00	38911009	491833015
813861000006112	Haemolytic anaemias	D1	D1...00	61261009	498963016
294448016	Polymorphonuclear neutrophil disorder NOS	D401z	D401z00	302874002	444861014
53913017	Idiopathic thrombocytopenic purpura	D3130	D313000	32273002	53913017
1227080015	Thrombocytopenia	42P2	42P2.00	302215000	1227080015
758831000006117	ITP - idiopathic thrombocytopenic purpura	D3130-2	D313012	32273002	3452119018
294144010	Other specified haemolytic anaemias	D1y	D1y..00	61261009	101791014
100341000006117	Thrombocytopenic eczema with immunodeficiency	C3912-1	C391211	36070007	60191017
294145011	Haemolytic anaemias NOS	D1z	D1z..00	61261009	498963016
454071000006113	Acquired haemolytic anaemias	D11	D11..00	4854004	495128014
786491000006113	Idiopathic neutropenia	D4000-1	D400011	248693006	371203012
294411013	Primary splenic neutropenia	D4001	D400100	191338000	294411013
257385013	Neutropenia	D400-2	D400.12	165517008	257385013
294376013	Other specified primary thrombocytopenia	D313y	D313y00	302873008	444860010
129661016	Thrombotic thrombocytopenic purpura	G7561	G756100	78129009	129661016
683051000006114	Myelodysplasia	B937W-1	B937W11	109995007	174702016
294593014	[X]Other autoimmune haemolytic anaemias	Dyu15	Dyu1500	413603009	2533077017
351323011	Hereditary thrombocytopenia NEC	D3131-1	D313111	267535004	399234016
351315014	Essential thrombocytopenia NOS	D313z-1	D313z11	267534000	399233010

821381000006117	Hereditary haemolytic anaemias	D10	D10..00	38911009	491833015
38970018	Transient neonatal thrombocytopenia	Q451	Q451.00	23205009	38970018
294094014	Primary warm-type haemolytic anaemia	D1101	D110100	191211009	294094014
293999013	Haemolytic anaemia due to hexokinase deficiency	D1030	D103000	191177007	293999013
500191000006115	Autoimmune haemolytic anaemias	D110	D110.00	413603009	2533078010
294375012	[X]Essential thrombocytopenia NOS	D3133	D313300	302215000	443796011
1490557013	Auto-immune thrombocytopenia	42P2-1	42P2.11	128091003	194685017
412281000006116	[X]Other nonthrombocytopenic purpura	Dyu31	Dyu3100	277791008	414503010
399233010	Primary thrombocytopenia	D313	D313.00	267534000	399233010
399235015	Primary thrombocytopenia NOS	D313z	D313z00	267534000	399233010
294112011	Other specified non-autoimmune haemolytic anaemia	D111y	D111y00	191216004	294104015
399243013	Acquired neutropenia NEC	D4008	D400800	303011007	445071015
294582012	[X]Haemolytic anaemias	Dyu1	Dyu1.00	61261009	498963016
294105019	Non-autoimmune haemolytic anaemia	D111	D111.00	191216004	294105019
675981000006110	Neutropenia	42J2	42J2.00	165517008	257385013
294389019	Thrombocytopenia NOS	D315	D315.00	302215000	1227080015
294007013	Enzyme deficiency haemolytic anaemia NOS	D103z	D103z00	38911009	65264014
294139015	Acquired haemolytic anaemia NOS	D11z	D11z.00	4854004	495128014
100361000006118	Thrombocytopenic purpura with absent radius	D3132	D313200	85589009	1235054010
453221019	Drug-induced autoimmune haemolytic anaemia	D1104	D110400	309742004	453221019
294102016	Autoimmune haemolytic anaemia NOS	D110z	D110z00	413603009	2533078010

293986010	Haemolytic anaemia due to glutathione metabolism disorder	D102	D102.00	191170009	293986010
294614011	[X]Other primary thrombocytopenia	Dyu32	Dyu3200	267534000	399233010
294085015	Hereditary haemolytic anaemia NOS	D10z	D10z.00	38911009	491833015
504771011	Drug-induced haemolytic anaemia	D1114	D111400	81711008	504771011
351137013	Acquired haemolytic anaemia with haemoglobinuria NEC	D112z-2	D112z12	191222008	294116014
399244019	Functional disorders of polymorphonuclear neutrophils	D401	D401.00	267543009	399244019
495896017	Microangiopathic haemolytic anaemia	D1111	D111100	51071000	495896017
6830410000 06112	Myelodysplasia	B937-4	B937.14	109995007	174702016
294385013	Other specified secondary thrombocytopenia	D314y	D314y00	74576004	123868015
294000010	Haemolytic anaemia due to pyruvate kinase deficiency	D1031	D103100	191178002	294000010
294111016	Infective haemolytic anaemia	D1115	D111500	38689004	491727016
399236019	Secondary thrombocytopenia NOS	D314z	D314z00	74576004	123868015
7866810000 06113	Idiopathic thrombocytopenic purpura	D313-2	D313.12	32273002	53913017
444860010	Thrombocytopenic purpura	D313-5	D313.15	302873008	444860010
294097019	Secondary cold-type haemolytic anaemia	D1102	D110200	191212002	294097019
294596018	[X]Other acquired haemolytic anaemias	Dyu17	Dyu1700	4854004	9091010
294595019	[X]Other nonautoimmune haemolytic anaemias	Dyu16	Dyu1600	191216004	294104015
294590012	[X]Other specified hereditary haemolytic anaemias	Dyu14	Dyu1400	38911009	65264014
1272366100 0006115	Acquired haemolytic anaemia NOS	^ESCT127 2366		597931000 000109	132480100000 0114
2576401000 006113	Acquired hemolytic anemia	^ESCTAC 257640		4854004	9091010

3712191000 006112	Acquired thrombocytopenia	^ESCTAC 371219		74576004	123868015
6844061000 006114	Autoimmune hemolytic anemia	^ESCTAU 684406		413603009	2533077017
4393301000 006114	Cold Donath Landsteiner type autoimmune haemolytic anaemia	^ESCTCO 439330		127057004	1783481014
4393351000 006113	Cold Donath Landsteiner type autoimmune hemolytic anemia	^ESCTCO 439335		127057004	1783852019
8138010000 06111	Haemolytic anaemia due glutathione metabolism disorder NOS	D102z	D102z00	191170009	293986010
8138210000 06118	Haemolytic anaemia due to glutathione metabolism disorder OS	D102y	D102y00	191170009	293986010
8138510000 06110	Haemolytic anaemia due triose phosphate isomerase deficiency	D1032	D103200	191179005	294003012
3495381000 006114	Hemolytic anemia	^ESCTHE 349538		61261009	101791014
4761321000 006115	Hemolytic anemia due to glutathione metabolism disorder	^ESCTHE 476132		191170009	293985014
4761351000 006112	Hemolytic anemia due to hexokinase deficiency	^ESCTHE 476135		191177007	293998017
4761371000 006119	Hemolytic anemia due to pyruvate kinase deficiency	^ESCTHE 476137		191178002	294001014
4761391000 006118	Hemolytic anemia due to triose phosphate isomerase deficiency	^ESCTHE 476139		191179005	294002019
1272365100 0006117	Hereditary haemolytic anaemia NOS	^ESCT127 2365		597871000 000103	132469100000 0115
3126221000 006116	Hereditary hemolytic anemia	^ESCTHE 312622		38911009	65264014
2584601000 006111	Hereditary nonspherocytic haemolytic anaemia (HNSHA) due to increased adenosine deaminase activity	^ESCTHE 258460		5315003	2620892014
2584611000 006114	Hereditary nonspherocytic hemolytic anemia (HNSHA) due to increased adenosine deaminase activity	^ESCTHE 258461		5315003	2620893016
2584621000 006118	Hereditary nonspherocytic hemolytic anemia due to	^ESCTHE 258462		5315003	2915153015

	increased adenosine deaminase activity				
3265001000 006116	Idiosyncratic neutropenia	^ESCTID3 26500		47318007	78893018
3019681000 006111	Immune thrombocytopenic purpura	^ESCTIM3 01968		32273002	3452118014
3019691000 006114	ITP - idiopathic thrombocytopenic purpura	^ESCTIT3 01969		32273002	301969100000 6114
1192631100 0006113	ITP - Idiopathic thrombocytopenic purpura	^ESCT119 2631		32273002	2972175010
3019701000 006114	ITP - immune thrombocytopenic purpura	^ESCTIT3 01970		32273002	3452120012
3327511000 006116	MAHA - Microangiopathic haemolytic anaemia	^ESCTMA 332751		51071000	1216697018
3327531000 006110	MAHA - Microangiopathic hemolytic anemia	^ESCTMA 332753		51071000	1218187018
294106018	Mechanical haemolytic anaemia	D1110	D111000	191217008	294106018
4761711000 006112	Mechanical hemolytic anemia	^ESCTME 476171		191217008	294107010
3327521000 006112	MHA - Microangiopathic haemolytic anaemia	^ESCTMH 332752		51071000	1216698011
3327541000 006117	MHA - Microangiopathic hemolytic anemia	^ESCTMH 332754		51071000	1218188011
3327461000 006119	Microangiopathic hemolytic anemia	^ESCTMI3 32746		51071000	85096017
5325991000 006118	Myelodysplasia of spinal cord	^ESCTMY 532599		253124001	2966572018
6830610000 06111	Myelodysplasia of spinal cord	P25y1-2	P25y112	253124001	377009014
4761691000 006114	Non-autoimmune hemolytic anemia	^ESCTNO 476169		191216004	294104015
293996018	Other enzyme deficiency haemolytic anaemia	D103	D103.00	38911009	65264014
4761611000 006116	Primary cold-type hemolytic anemia	^ESCTPR 476161		191210005	294092013
3957381000 006114	Primary neutropenia	^ESCTPR 395738		89655007	508614010

4761631000006110	Primary warm-type hemolytic anemia	^ESCTPR476163		191211009	294095010
4761651000006115	Secondary cold-type hemolytic anemia	^ESCTSE476165		191212002	294096011
294098012	Secondary warm-type haemolytic anaemia	D1103	D110300	191213007	294098012
4761671000006113	Secondary warm-type hemolytic anemia	^ESCTSE476167		191213007	294099016
5904291000006111	Thrombocytopenic disorder	^ESCTTH590429		302215000	443796011
3770811000006115	TTP - Thrombotic thrombocytopenic purpura	^ESCTTT377081		78129009	503764013
2767385013	Chronic kidney disease stage 4	K054	K054.00	431857002	2767385013
304091000000116	Chronic kidney disease stage 4	1Z13	1Z13.00	431857002	2767385013
596401000000111	Chronic kidney disease stage 5 without proteinuria	1Z1L	1Z1L.00	324541000000105	596401000000111
596321000000110	Chronic kidney disease stage 5 with proteinuria	1Z1K	1Z1K.00	324501000000107	596321000000110
304111000000114	Chronic kidney disease stage 5	1Z14	1Z14.00	433146000	2767154014
596191000000110	Chronic kidney disease stage 4 with proteinuria	1Z1H	1Z1H.00	324441000000106	596191000000110
596261000000111	Chronic kidney disease stage 4 without proteinuria	1Z1J	1Z1J.00	324471000000100	596261000000111
2767154014	Chronic kidney disease stage 5	K055	K055.00	433146000	2767154014
994431000006114	Chronic kidney disease stage 4	EMISNQCH21		994431000006105	994431000006114
994441000006116	Chronic kidney disease stage 5	EMISNQCH22		994441000006100	994441000006116
8346101000006114	Chronic kidney disease with glomerular filtration rate category G4 and albuminuria category A1	^ESCTCH834610		950181000000106	2428071000000110
8346121000006116	Chronic kidney disease with glomerular filtration rate category G4 and albuminuria category A2	^ESCTCH834612		950211000000107	2428121000000110

8346151000006113	Chronic kidney disease with glomerular filtration rate category G4 and albuminuria category A3	^ESCTCH834615		950231000000104	2428171000000114
8346171000006115	Chronic kidney disease with glomerular filtration rate category G5 and albuminuria category A1	^ESCTCH834617		950251000000106	2428261000000114
8346201000006116	Chronic kidney disease with glomerular filtration rate category G5 and albuminuria category A2	^ESCTCH834620		950291000000103	2428311000000119
8346221000006114	Chronic kidney disease with glomerular filtration rate category G5 and albuminuria category A3	^ESCTCH834622		950311000000102	2428361000000117
8104451000006111	CKD (chronic kidney disease) stage 4 with proteinuria	^ESCTCK810445		324441000000106	1693001000000116
8104481000006115	CKD (chronic kidney disease) stage 4 without proteinuria	^ESCTCK810448		324471000000100	1693051000000115
8104511000006111	CKD (chronic kidney disease) stage 5 with proteinuria	^ESCTCK810451		324501000000107	1693061000000117
8104551000006112	CKD (chronic kidney disease) stage 5 without proteinuria	^ESCTCK810455		324541000000105	1692911000000112
3245881000006114	End stage chronic renal failure	^ESCTEN324588		46177005	2647096016
3245861000006116	ESCRF - End stage chronic renal failure	^ESCTES324586		46177005	1230315018
8346121000006116	Chronic kidney disease stage 4	^ESCTCH834612		950211000000107	2428121000000110
8346151000006113	Chronic kidney disease stage 4	^ESCTCH834615		950231000000104	2428171000000114
8346171000006115	Chronic kidney disease stage 5	^ESCTCH834617		950251000000106	2428261000000114
8346201000006116	Chronic kidney disease stage 5	^ESCTCH834620		950291000000103	2428311000000119
8346221000006114	Chronic kidney disease stage 5	^ESCTCH834622		950311000000102	2428361000000117
1940501000006119	CKD with GFR category G4 & albuminuria category A1	1Z1a	1Z1a.00	950181000000106	2428021000000111
1940511000006116	CKD with GFR category G4 & albuminuria category A2	1Z1b	1Z1b.00	950211000000107	2428091000000114

1940521000 006112	CKD with GFR category G4 & albuminuria category A3	1Z1c	1Z1c.00	950231000 000104	242814100000 0115
1940531000 006110	CKD with GFR category G5 & albuminuria category A1	1Z1d	1Z1d.00	950251000 000106	242819100000 0113
1940541000 006117	CKD with GFR category G5 & albuminuria category A2	1Z1e	1Z1e.00	950291000 000103	242828100000 0117
1940551000 006115	CKD with GFR category G5 & albuminuria category A3	1Z1f	1Z1f.00	950311000 000102	242833100000 0110
